# Effects of Non‐Pharmacological Interventions on the Swallowing Function of Patients With Post‐Stroke Dysphagia: A Systematic Review and Network Meta‐Analysis

**DOI:** 10.1111/joor.13901

**Published:** 2024-11-12

**Authors:** Bohan Zhang, Ka Po Wong, Cai Guo, Shu‐Cheng Chen, Shuojin Fu, Ruifu Kang, Qian Xiao, Jing Qin

**Affiliations:** ^1^ Centre for Smart Health, School of Nursing The Hong Kong Polytechnic University Kowloon Hong Kong China; ^2^ Department of Applied Social Sciences The Hong Kong Polytechnic University Kowloon Hong Kong China; ^3^ School of Computing and Information Engineering Hanshan Normal University Chaozhou Guangdong China; ^4^ School of Nursing Capital Medical University Beijing China

**Keywords:** deglutition disorders, dysphagia, network meta‐analysis, post‐stroke, rehabilitation, swallowing

## Abstract

**Background:**

Post‐stroke dysphagia can lead to serious complications and appropriate rehabilitation can significantly improve swallowing function. However, the best rehabilitation method for post‐stroke dysphagia patients is not clear at the present stage, so it is necessary to conduct a comprehensive network meta‐analysis and systematic review of different interventions for dysphagia.

**Objective:**

To compare the effectiveness and ranking of different interventions for improving swallowing function, and feeding and daily function in patients with post‐stroke dysphagia.

**Methods:**

Seven databases were searched from the date of inception to September 1, 2022. Two investigators independently conducted literature searches, selected randomized controlled trials on dysphagia interventions, and assessed study quality. Network meta‐analysis was conducted by using Stata software.

**Results:**

A total of 33 studies involving 1,341 patients were included. According to the ranking probabilities, acupuncture was rated as the most effective of all interventions to enhance patients' swallowing function (surface under cumulative ranking curve values [SUCRCV]: 99.0%, standardized mean difference [SMD]: −2.40, 95% confidence interval [CI]: −3.38 to −1.43), followed by the chin tuck against resistance exercise (CTAR, SUCRA: 89.9%, SMD: −1.83, 95% CI: −2.69 to −0.97). Among all the interventions, acupuncture was the most effective for feeding and daily function (SUCRCV: 88.4%, SMD: −1.62, 95% CI: −2.94 to −0.30).

**Conclusions:**

The results showed that acupuncture was the most effective in the rehabilitation of patients with post‐stroke dysphagia, followed by CTAR. Considering that CTAR is a low‐cost and highly feasible intervention, we suggest that CTAR should be selected as a rehabilitation measure for patients with post‐stroke dysphagia to improve their swallowing function.

## Graphical abstract

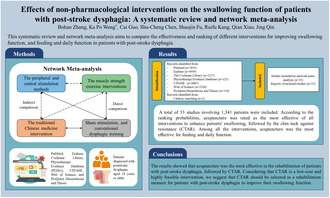

## 1 Introduction

Dysphagia is a clinical condition characterised by structural and/or functional impairments of the jaw, lips, tongue, soft palate, pharynx, oesophagus, and other organs, which hinder the safe and effective delivery of food from the mouth to the stomach [1]. According to a meta‐analysis published in 2022, dysphagia affects up to two‐thirds of stroke patients worldwide. Among patients with early dysphagia following a stroke, at least 75% experience moderate to severe symptoms [2]. Post‐stroke dysphagia results in impaired swallowing function, which consequently leads to compromised feeding abilities and potentially weakened immune responses, ultimately affecting patients' daily functioning [3]. Dysphagia can lead to reduced patients' immune response, prolonged hospitalisation, increased mortality and higher hospitalisation costs [4]. Notably, the risk of death in stroke patients with dysphagia is 8.5 times higher than in patients without dysphagia, with a 30‐day mortality rate ranging from 13.8% to 40% [5].

There are evidence that swallowing therapy can be effective in improving swallowing ability, shortening inpatient stay, and reducing the incidence of pneumonia [6, 7]. However, swallowing is a complex process involving the bilateral cerebral cortex, the central pattern generator, five cranial nerves, and 26 pairs of muscles [8]. It is found that only 17.6% of patients returned to their pre‐stroke diet after receiving the usual care [9]. Therefore, effective swallowing rehabilitation methods are needed to improve the swallowing function and feeding and daily function of patients with stroke.

Recent research has explored diverse approaches to facilitate recovery from post‐stroke dysphagia, which can be categorised into three main domains [6]: (1) peripheral and central stimulation methods, (2) swallowing exercises focusing on muscle strength, and (3) traditional Chinese medicine. The peripheral and central stimulation methods include neuromuscular electrical stimulation (NMES), intermittent theta burst stimulation (iTBS), transcranial direct current stimulation (tDCS), repetitive transcranial magnetic stimulation (rTMS), and pharyngeal electric stimulation (PES). Swallowing muscle strength exercises encompass various techniques, including tongue‐pressure resistance training, chin tuck against resistance exercise (CTAR), shaker exercise, and expiratory muscle strength training (EMST). In the realm of traditional Chinese medicine, acupuncture is frequently employed for swallowing rehabilitation. However, current treatment methods for post‐stroke dysphagia face several limitations. Swallowing muscle strength training and traditional Chinese medicine often require several weeks of continuous treatment to achieve clinical effects. Conversely, electrical stimulation techniques require substantial professional assistance and specialised rehabilitation equipment [10]. These limitations may result in suboptimal patient adherence to treatment protocols and potentially compromise overall therapeutic outcomes [11].

Several studies have focused on the effect of several rehabilitation interventions to improve swallowing function in post‐stroke patients; however, their results have been inconsistent and the specific effects remain unclear. Lim et al. [12] compared the effectiveness between conventional dysphagia training, rTMS and NMES, and found that both rTMS and NMES were more effective in improving swallowing function, but not feeding function in post‐stroke dysphagia compared to conventional rehabilitation. Additionally, a randomized controlled trial (RCT) by Xie et al. [13] found no significant difference between rTMS and iTBS in improving post‐stroke swallowing function. Huang et al. [14] revealed that both acupuncture and electrical stimulation were more effective than conventional treatment in improving swallowing function, with comparable efficacy between the two. In contrast, a systematic review [15] showed that respiratory muscle strength training and acupuncture were effective in treating dysphagia and enhancing daily function in post‐stroke patients. However, there was insufficient evidence for a therapeutic effect of electrical stimulation on dysphagia. Understanding the most effective ways to recover from dysphagia will assist physicians in making clinical decisions, enable nurses to develop care plans, and provide supportive counselling to patients and their families [16]. Therefore, a comprehensive comparison of multiple interventions is necessary to develop evidence‐based care strategies aimed at improving swallowing function in patients after stroke.

Conventional paired meta‐analysis involves a direct comparison between only two interventions and cannot simultaneously estimate the relative effectiveness of all available interventions [17]. Although published meta‐analyses have demonstrated the beneficial effects of interventions such as tDCS [18], rTMS [19], and exercise therapy [20] on swallowing function, feeding abilities, and daily function, a comprehensive quantitative comparison of the three major intervention categories (peripheral and central stimulation methods, swallowing muscle strength exercises and traditional Chinese medicine) for post‐stroke dysphagia remains lacking. Network meta‐analysis facilitates both direct and indirect comparisons based on logical reasoning, allowing for the generation of a rank order of all interventions and the identification of the most effective interventions [21]. Therefore, we conducted a network meta‐analysis to evaluate the comparative effectiveness and ranking of all swallowing therapy interventions in improving swallowing function and feeding and daily function in patients with post‐stroke.

## 2 Methods

### 2.1 Registration

A network meta‐analysis combining direct and indirect evidence was performed, adhering to the Preferred Reporting Items for Systematic Reviews and Meta‐analysis network statement for reporting of systematic reviews incorporating network meta‐analysis (Supporting Information S1) [22]. The protocol for this study was registered with PROSPERO (registration number: CRD42023391951).

### 2.2 Search Strategy

A literature search was conducted across seven electronic databases: PubMed, Embase, Cochrane Library, Physiotherapy Evidence Database (PEDro), CINAHL, Web of Science, and ProQuest Dissertations and Theses. The search encompassed all available literature from the inception of each database through September 1, 2022. The search terms used were (stroke) AND (dysphagia OR deglutition disorders OR swallowing disorder). A detailed Pubmed search strategy was provided in Supporting Information S2. In addition, we manually searched the reference lists of these relevant studies to identify eligible articles for inclusion in the analysis.

### 2.3 Inclusion and Exclusion Criteria

The inclusion criteria were as follows: (1) Participants: patients diagnosed with poststroke dysphagia, aged 18 years or older; (2) Interventions: interventions were related to swallowing rehabilitation, without limitations on type. The peripheral and central stimulation methods were neuromuscular electrical stimulation (NMES), intermittent theta burst stimulation (iTBS), transcranial direct current stimulation (tDCS), repetitive transcranial magnetic stimulation (rTMS), and pharyngeal electric stimulation (PES). The muscle strength exercise interventions were tongue‐pressure resistance training (TPRT), chin tuck against resistance exercise (CTAR), shaker exercise, and expiratory muscle strength training (EMST). The traditional Chinese medicine intervention was acupuncture; (3) Controls: using sham stimulation, no stimulation, and conventional dysphagia training (CDT), such as effortful swallow, other swallow movements, or compensatory interventions, as the control group; (4) Outcomes: the primary outcome was swallowing function assessed before and after the intervention. All validated quantitative scores measuring clinical or radiological swallowing function in patients with dysphagia were acceptable, such as the Penetration Aspiration Scale, water‐swallowing test, and videofluoroscopic swallowing study. Secondary outcomes were feeding and daily function assessed before and after the intervention, such as the swallowing quality of life questionnaire, Barthel index, and Functional Oral Intake Scale; and (5) randomized controlled trials (RCTs).

The exclusion criteria were: (1) duplicate records; (2) animal studies or bench studies; (3) incomplete and inaccessible information for network meta‐analyses data analysis, such as reviews, abstracts, and research protocols; and (4) studies not published in English.

After excluding duplicate literature, potentially eligible articles were screened by title and abstract. Only articles that met the inclusion criteria were included in the final analysis. The literature search and selection were conducted independently by two researchers (B.Z., G.C.), with disagreements resolved by consensus or with the involvement of a third researcher (K.P.W.).

### 2.4 Data Extraction

Relevant data were collected independently by two reviewers (B.Z., G.C.) using a pre‐designed data extraction sheet. Data were extracted from the included studies, which comprised authors' names, year of publication, sample size, time of onset, gender, age, intervention, intervention intensity, intervention frequency, assessment tools, outcomes and adverse events. Any disagreements were resolved through discussion and a consensus was reached.

### 2.5 Risk of Bias

Two evaluators (B.Z., G.C.) independently examined the risk of bias of the included studies using the Cochrane Risk of Bias Tool 2.0 (RoB2) [23]. The assessed domains included randomization, intervention assignment, adherence to interventions, missing outcome data, measurement of outcomes, reporting selection of outcomes and overall bias. The overall risk of bias was classified as “high risk,” “low risk” or “some concern”. Any discrepancies were resolved with the involvement of a third researcher (K.P.W.) and a consensus was reached.

### 2.6 Data Analysis

Due to the differences in results among the various scales, standardized mean differences (SMD) were used to compare the outcomes of the different interventions [24]. SMD cannot correct for variations in scale orientation; in our study, four studies [25, 26, 27, 28] used a scale where scores decreased with the severity of swallowing function, while the other studies used a scale that increased. Therefore, the mean of these four studies was subtracted from the maximum possible value of the scale to ensure that all scales were pointing in the same direction before standardization [29]. The frequency method and multivariate random effects meta‐regression models were used for network meta‐analysis, implemented through the Network Command Suite in Stata 17.0.

Paired meta‐analysis was performed using a random effects model to directly compare the treatment effects of any two interventions. The Cochrane *Q* test (*p* < 0.1) and *I* ^2^ statistic (> 50%) were used to test for between‐study heterogeneity [30]. Depending on the value of *I* ^2^, heterogeneity was classified as unimportant (0% to 30%), moderate (30% to 50%), substantial (50% to 75%), or considerable (75% to 100%).

A network geometry plot was used to illustrate the evidence in the network regarding the swallowing function and daily feeding function. In this plot, the size of each nodes was directly proportional to the number of participants associated with that intervention method, while the thickness of the connecting lines between the node was directly proportional to the number of participants in the trial directly comparing the two intervention methods [31].

The treatment effects of the interventions were ranked using the surface under the cumulative ranking curve (SUCRA), with SUCRA values ranging from 1 to 0. The higher the SUCRA score, the larger the area under the curve, indicating a higher likelihood that the intervention method was the best [32].

Paired meta‐analysis and network meta‐analysis were conducted to compare the effects between interventions and controls. All results were reported as standardized mean differences (SMD) and 95% confidence intervals (CI), indicating that the results were not statistically different when the 95% CI included 0. A league table was used to report these results.

Inconsistency between direct and indirect effects was assessed using loop‐specific inconsistency and the side‐splitting models [33]. To test for potential publication bias, Egger's test was used [34]. Sensitivity analysis was used to test the robustness of the study results and to detect whether any particular study resulted in a large proportion of heterogeneity.

## 3 Results

### 3.1 Literature Screening Process and Results

**FIGURE 1:**
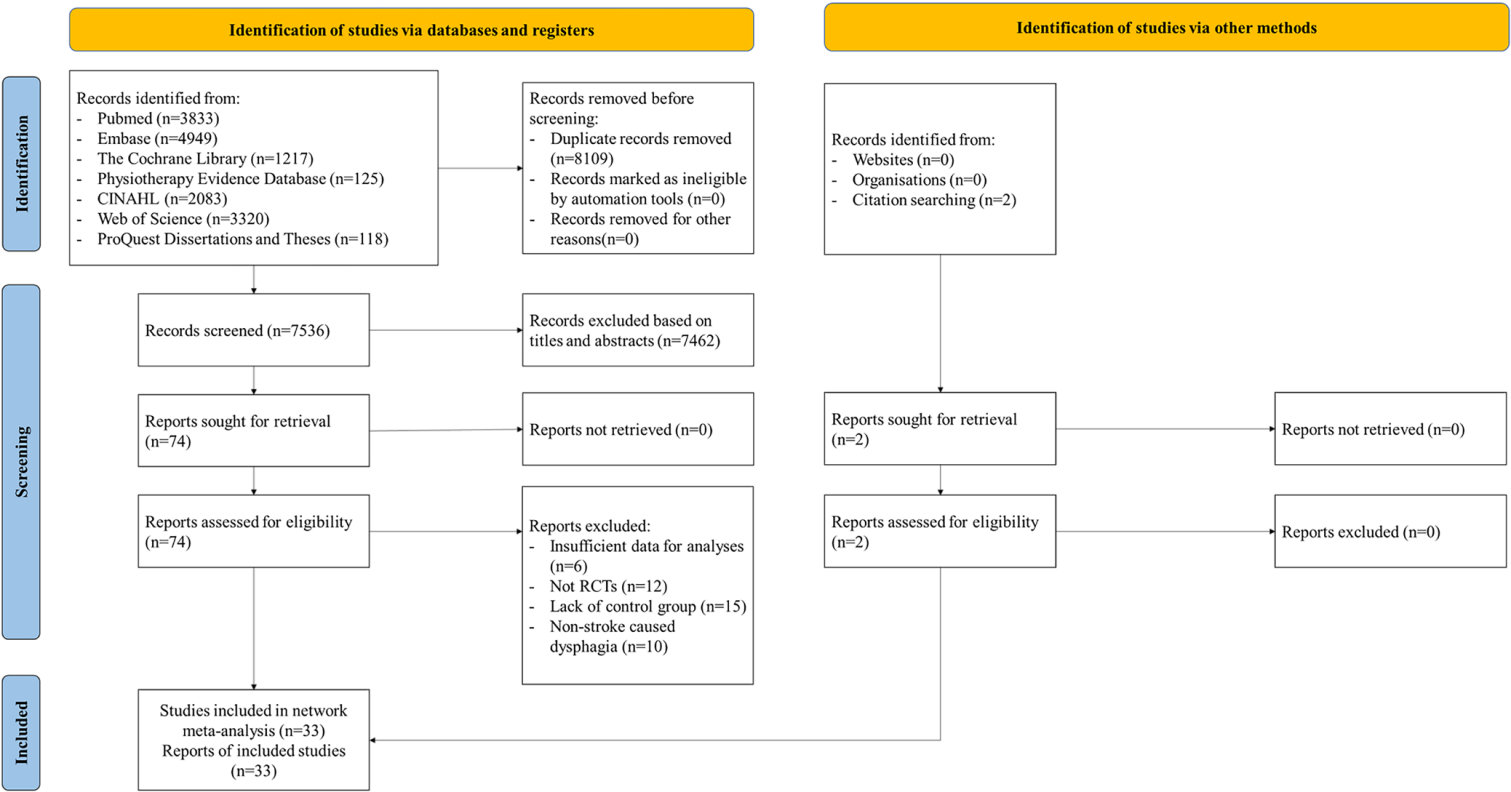
Literature screening process and results.

A total of 15,645 articles were searched through the electronic databases. After removing 8,109 duplicates, we screened the remaining articles by title and abstract, excluding 7,462 articles that did not meet the inclusion criteria. The remaining 74 full‐text articles were further examined and 43 articles were excluded due to non‐RCT design, lack of a control group (e.g., studies comparing different stimulation locations or intensities, without a standard care or placebo control group), or non‐stroke‐caused dysphagia. In addition, we identified relevant studies from the reference list of included studies, resulting in the inclusion of 33 RCTs for network meta‐analysis (Figure 1).

### 3.2 Description of Included Studies

A total of 33 RCTs were published between 2013 and 2022, involving 1,341 patients with stroke, including 795 males and 558 females, with ages ranging from 55.3 to 79 years. Among these patients, 630 had ischemic stroke and 266 had hemorrhagic stroke, with stroke onset times ranging from 4.1 days to 17.3 months. Nine countries were involved in the publication of these studies, with Korea publishing the most (*n* = 16), followed by China (*n* = 7), Turkey (*n* = 2), UK (*n* = 2), Iran (*n* = 2), USA (*n* = 1), Spain (*n* = 1), Italy (*n* = 1) and Germany (*n* = 1). The sample size ranged between 87 to 98. The details of the characteristics of the included studies are shown in Supporting Information S3.

A total of 12 types of interventions were reported across all trials, including neuromuscular electrical stimulation (NMES, *n* = 7), intermittent theta burst stimulation (iTBS, *n* = 2), transcranial direct current stimulation (tDCS, *n* = 4), repetitive transcranial magnetic stimulation (rTMS, *n* = 4), and pharyngeal electric stimulation (PES, *n* = 1) for peripheral and central stimulation methods; the muscle strength exercise interventions were tongue‐pressure resistance training (TPRT, *n* = 4), chin tuck against resistance exercise (CTAR, *n* = 6), shaker exercise (*n* = 4), and expiratory muscle strength training (EMST, *n* = 4). The traditional Chinese medicine intervention was acupuncture (*n* = 1); sham control (*n* = 12) and conventional dysphagia training (CDT, *n* = 18). The duration of the intervention ranged from 3 days to 8 weeks. One of the studies included three intervention groups [35], while the others had only two intervention groups.

### 3.3 Risk of Bias

In terms of the overall risk of bias, three studies were rated as low risk, 19 studies as high risk and 11 as having some concern (Supporting Information S4). Although all included studies were RCTs, eight studies (24.2%) did not report the methodology used for randomization. Only 13 studies (39.4%) completed allocation concealment. Participants were blinded in 11 studies (33.3%), while assessor blinding was achieved in 21 studies. Three studies (9.1%) mentioned single blinding but did not identify who was blinded. Only one study achieved blinding for participants, deliverers and assessors. All studies had reasonable outcome assessment indicators, consistent and comparable baseline, complete reporting of data and no concealment of results.

### 3.4 Network Diagram

**FIGURE 2:**
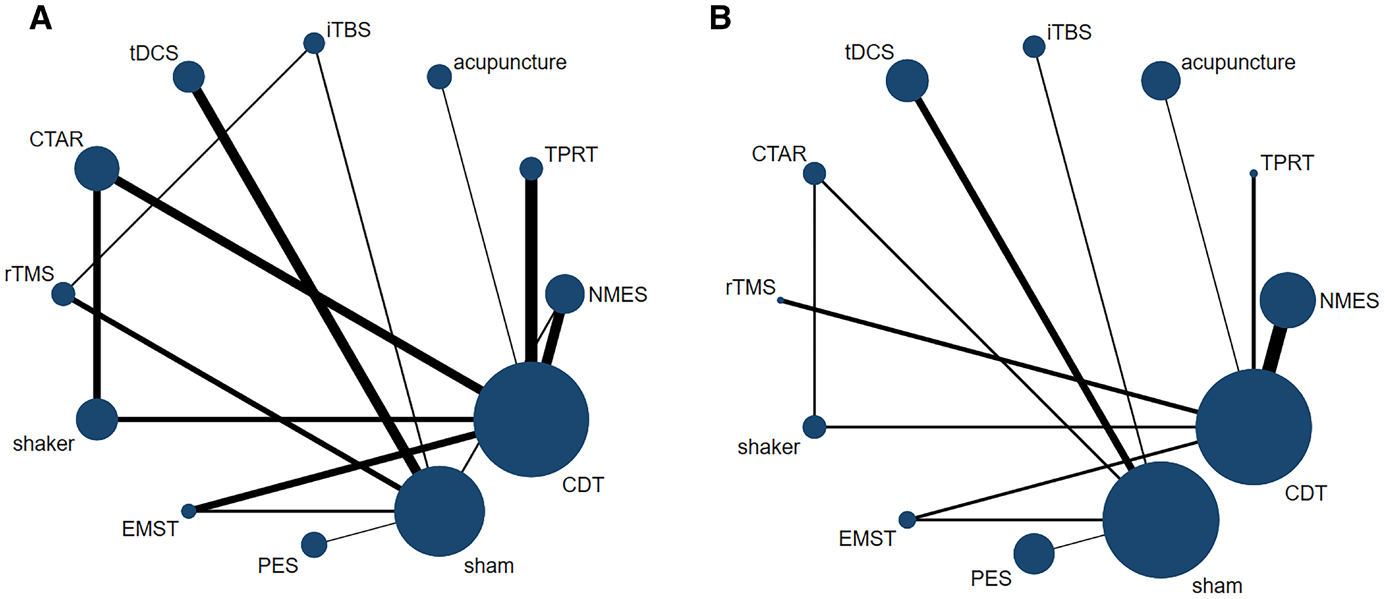
Network plots of the comparison of all interventions. (A) Swallowing function; (B) Feeding and daily function. Neuromuscular electrical stimulation (NMES), Intermittent theta burst stimulation (iTBS), Transcranial direct current stimulation (tDCS), Repetitive transcranial magnetic stimulation (rTMS), Pharyngeal electric stimulation (PES), Tongue‐pressure resistance training (TPRT), Chin tuck against resistance exercise, Expiratory muscle strength training (EMST). * The size of a node was directly proportional to the number of participants in that node for the intervention method and the thickness of the connecting line connecting the node was directly proportional to the number of participants in the trial directly comparing the two intervention methods.

Network plots of swallowing function, and feeding and daily function from the 33 included studies are shown in Figure 2. For swallowing function (Figure 2A), 12 interventions across 28 trials involving 1,189 participants formed a star‐shaped network plot. Of these interventions, CDT had the largest sample size (279 participants), followed by sham control (240 participants), NMES (100 participants), CTAR (85 participants), tDCS (78 participants), shaker (77 participants), PES (70 participants), acupuncture (60 participants), iTBS (57 participants), TPRT (56 participants), rTMS (55 participants) and EMST (32 participants). Two closed loops were formed in the network analysis, so inconsistency tests were performed in both loops. In terms of feeding and daily function (Figure 2B), 12 interventions were connected through CDT and sham control, forming a star network map that included a total of 18 trials with 791 participants.

### 3.5 Inconsistency Test

The inconsistency test was conducted using loop‐specific heterogeneity estimates and the side‐splitting models, and no significant differences were found (all *p* > 0.05) (Supporting Information S4).

### 3.6 Paired Meta‐Analysis

In swallowing function, the results of a paired meta‐analysis between iTBS, NMES, EMST and sham control showed that iTBS, NMES and EMST significantly improved swallowing function in patients with stroke (iTBS: SMD = 0.87, 95% CI: 0.35–1.38, *I* ^2^ = 0.0%, indicating no heterogeneity; NMES: SMD = 1.07, 95% CI: 0.47–1.67, *I* ^2^ = 0.0%, indicating no heterogeneity; EMST: SMD = 0.96, 95% CI: 0.14–1.78, *I* ^2^ = 0.0%, indicating no heterogeneity). The paired meta‐analysis comparing CTAR, shaker, acupuncture, and CDT showed that the efficacy of CTAR, shaker and acupuncture was significantly better than that of CDT (CTAR: SMD = 1.02, 95% CI: 0.43–1.62, *I* ^2^ = 45.0%, indicating no heterogeneity; shaker: SMD = 1.01, 95% CI: 0.29–1.74, *I* ^2^ = 57.4%, indicating no heterogeneity; acupuncture: SMD = 1.73, 95% CI: 1.31–2.15, *I* ^2^ = 0.0%, indicating no heterogeneity) (Supporting Information S4).

The paired meta‐analysis found that iTBS and tDCS were more effective in enhancing patients' feeding and daily function compared to sham control (iTBS: SMD = 1.21, 95% CI: 0.67–1.74, *I* ^2^ = 0.0%, indicating no heterogeneity; tDCS: SMD = 0.59, 95% CI: −0.06 to −1.23, *I* ^2^ = 68.2%, indicating no heterogeneity). Compared with CDT, acupuncture and shaker demonstrated a more significant effect on the feeding and daily function of stroke patients with dysphagia (acupuncture: SMD = 1.62, 95% CI: 1.21–2.04, *I* ^2^ = 0.0%, indicating no heterogeneity; shaker: SMD = 0.87, 95% CI: 0.13–1.61, *I* ^2^ = 0.0%, indicating no heterogeneity) (Supporting Information S4).

### 3.7 Network Meta‐Analysis and Probability Ranking of the Effects of Swallowing Function

**FIGURE 3:**
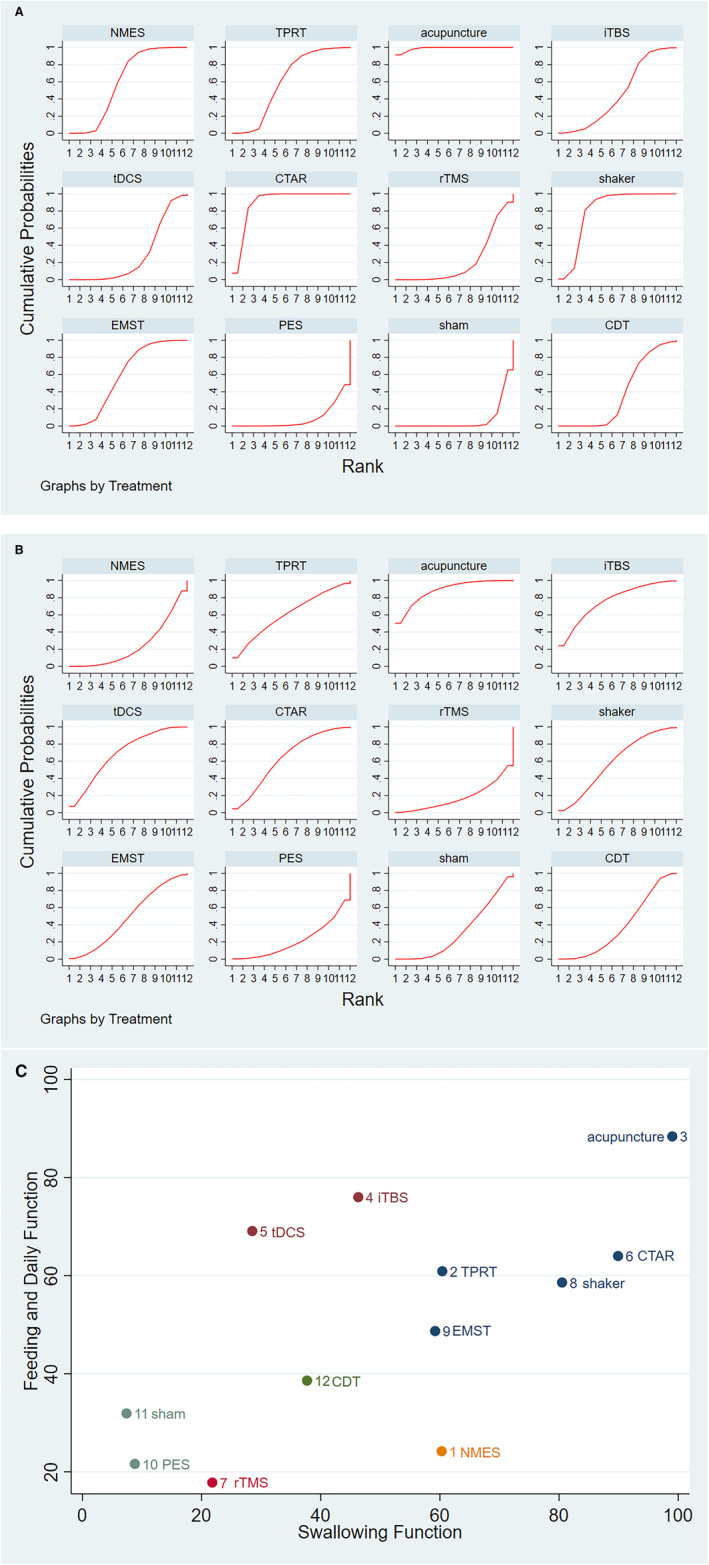
The probability ranking of the effects of all interventions. (A) SUCRA of swallowing function; (B) SUCRA of feeding and daily function; (C) Ranking results for the combination of swallowing function and feeding and daily function. Neuromuscular electrical stimulation (NMES), Intermittent theta burst stimulation (iTBS), Transcranial direct current stimulation (tDCS), Repetitive transcranial magnetic stimulation (rTMS), Pharyngeal electric stimulation (PES), Tongue‐pressure resistance training (TPRT), Chin tuck against resistance exercise, Expiratory muscle strength training (EMST).

The network meta‐analysis showed that acupuncture (SUCRA: 99.0%, SMD: −2.40, 95% CI: −3.38 to −1.43), CTAR (SUCRA: 89.9%, SMD: −1.83, 95% CI: −2.69 to −0.97) and shaker (SUCRA: 80.5%, SMD: −1.57, 95% CI: −2.45 to −0.70) were significantly better than sham control in improving swallowing function in patients with stroke dysphagia. This was followed by TPRT(SUCRA: 60.4%, SMD: −1.06, 95% CI: −1.9 to −0.20), NMES (SUCRA: 60.3%, SMD: −1.03, 95% CI: −1.07 to −0.36), EMST (SUCRA: 59.2%, SMD: −1.02, 95% CI: −1.77 to −0.28) and iTBS (SUCRA: 46.3%, SMD: −0.72, 95% CI: −1.32 to −0.11) (Table 1 lower left triangle, Figure 3A).

**TABLE 1:** Network analysis of swallowing function, feeding and daily function.

NMES	0.96 (−0.78, 2.70)	1.94 (0.47, 3.42)	1.41 (−0.80, 3.62)	1.11 (−0.80, 3.03)	0.98 (−0.73, 2.70)	−0.46 (−2.32, 1.39)	0.85 (−0.61, 2.30)	0.57 (−0.92, 2.06)	−0.22 (−2.39, 1.95)	0.21 (−1.53, 1.94)	0.32 (−0.33,0.97)
0.03 (−0.59, 0.64)	TPRT	0.98 (−1.10, 3.07)	0.45 (−2.21, 3.11)	0.15 (−2.27, 2.58)	0.02 (−2.24, 2.29)	−1.42 (−3.79, 0.95)	−0.11 (−2.19, 1.96)	−0.39 (−2.49, 1.71)	−1.18 (−3.81, 1.45)	−0.75 (−3.04, 1.53)	−0.64 (−2.25, 0.98)
1.37 (0.60, 2.15)	1.35 (0.54, 2.15)	Acupuncture	−0.53 (−3.02, 1.96)	−0.83 (−3.07, 1.41)	−0.96 (−3.03, 1.11)	−2.41 (−4.59, −0.22)	−1.10 (−2.95, 0.76)	−1.37 (−3.26, 0.52)	−2.16 (−4.62, 0.30)	−1.74 (−3.82, 0.35)	−1.62 (−2.94, −0.30)
−0.31 (−1.22, 0.59)	−0.34 (−1.39, 0.71)	−1.69 (−2.84, −0.54)	iTBS	−0.30 (−1.89, 1.29)	−0.43 (−2.32, 1.46)	−1.88 (−4.61, 0.86)	−0.57 (−2.66, 1.53)	−0.84 (−2.74, 1.06)	−1.63 (−3.52, 0.25)	−1.21 (−2.57, 0.16)	−1.09 (−3.21, 1.02)
−0.65 (−1.44, 0.14)	−0.68 (−1.63, 0.27)	−2.02 (−3.08, −0.97)	−0.34 (−1.07, 0.40)	tDCS	−0.13 (−1.67, 1.41)	−1.58 (−4.08, 0.93)	−0.27 (−2.06, 1.52)	−0.54 (−2.09, 1.01)	−1.34 (−2.87, 0.20)	−0.91 (−1.72, −0.09)	−0.79 (−2.60, 1.02)
0.80 (0.17, 1.42)	0.77 (0.11, 1.43)	−0.58 (−1.39, 0.24)	1.11 (0.06, 2.16)	1.45 (0.49, 2.40)	CTAR	−1.45 (−3.80, 0.91)	−0.14 (−1.41, 1.14)	−0.41 (−2.02, 1.20)	−1.20 (−3.05, 0.64)	−0.78 (−2.08, 0.53)	−0.66 (−2.26, 0.93)
−0.77 (−1.63, 0.09)	−0.80 (−1.81, 0.22)	−2.15 (−3.26, −1.03)	−0.46 (−1.06, 0.15)	−0.12 (−0.80, 0.56)	−1.57 (−2.59, −0.55)	rTMS	1.31 (−0.86, 3.48)	1.03 (−1.16, 3.23)	0.24 (−2.47, 2.95)	0.67 (−1.70, 3.04)	0.78 (−0.95, 2.52)
0.54 (−0.10, 1.18)	0.52 (−0.16, 1.19)	−0.83 (−1.66, −0.00)	0.86 (−0.21, 1.92)	1.19 (0.23, 2.16)	−0.25 (−0.69, 0.18)	1.31 (0.29, 2.34)	Shaker	−0.27 (−1.89, 1.34)	−1.07 (−3.13, 0.99)	−0.64 (−2.23, 0.95)	−0.52 (−1.83, 0.78)
−0.01 (−0.72, 0.71)	−0.03 (−0.84, 0.77)	−1.38 (−2.31, −0.45)	0.31 (−0.65, 1.27)	0.64 (−0.21, 1.50)	−0.80 (−1.61, 0.00)	0.76 (−0.16, 1.69)	−0.55 (−1.37, 0.27)	EMST	−0.79 (−2.65, 1.06)	−0.37 (−1.68, 0.95)	−0.25 (−1.60, 1.10)
−1.08 (−1.99, −0.17)	−1.11 (−2.16, −0.05)	−2.45 (−3.61, −1.30)	−0.77 (−1.63, 0.10)	−0.43 (−1.17, 0.31)	−1.88 (−2.94, −0.82)	−0.31 (−1.13, 0.51)	−1.62 (−2.69, −0.55)	−1.07 (−2.04, −0.11)	PES	0.43 (−0.88, 1.73)	0.54 (−1.53, 2.62)
−1.03 (−1.70, −0.36)	−1.06 (−1.91, −0.20)	−2.40 (−3.38, −1.43)	−0.72 (−1.32, −0.11)	−0.38 (−0.79, 0.04)	−1.83 (−2.69, −0.97)	−0.26 (−0.80, 0.28)	−1.57 (−2.45, −0.70)	−1.02 (−1.77, −0.28)	0.05 (−0.57, 0.67)	Sham	0.11 (−1.50, 1.73)
−0.36 (−0.76, 0.04)	−0.38 (−0.85, 0.08)	−1.73 (−2.39, −1.07)	−0.04 (−0.98, 0.89)	0.29 (−0.54, 1.12)	−1.16 (−1.63, −0.68)	0.41 (−0.48, 1.31)	−0.90 (−1.40, −0.40)	−0.35 (−1.01, 0.31)	0.72 (−0.23, 1.67)	0.67 (−0.05, 1.39)	CDT

*Note:* The lower left triangle gives the results of the swallowing function network meta‐analysis and the upper right triangle gives the results of the feeding and daily function network meta‐analysis.

Abbreviations: CDT, Conventional dysphagia training; CTAR, Chin Tuck against resistance exercise; EMST, Expiratory muscle strength training; iTBS, Intermittent theta burst stimulation; NMES, Neuromuscular electrical stimulation; PES, Pharyngeal electric stimulation; rTMS, Repetitive transcranial magnetic stimulation; tDCS, Transcranial direct current stimulation; TPRT, Tongue‐pressure resistance training.

Compared with CDT, acupuncture (SMD: −1.73, 95% CI: −2.39 to −1.07) significantly improved swallowing function in patients with stroke dysphagia, followed by CTAR (SMD: −1.16, 95% CI: −1.63 to −0.68) and shaker (SMD: −0.90, 95% CI: −1.40 to −0.40) (Table 1 lower left triangle).

### 3.8 Network Meta‐Analysis and Probability Ranking of the Effects of Feeding and Daily Function

The results of the network meta‐analysis suggested that acupuncture (SMD: −1.62, 95% CI: −2.94 to −0.30) significantly enhanced the recovery of patients' feeding and daily function compared to conventional CDT, ranking highest according to the SUCRA results (88.4%). This was followed by iTBS (SUCRA: 76.0%), tDCS (SUCRA: 69.1%) and CTAR (SUCRA: 64.0%). However, there were no significant differences in the effects of other interventions compared to sham control and CDT on improving feeding and daily function (Table 1 upper right triangle, Figure 3B).

As shown in Figure 3C, when combining the effects on swallowing function and feeding and daily function, acupuncture ranked as the most effective, followed by CTAR and shaker, while PES ranked the lowest.

### 3.9 Safety

An evaluation of the safety of different interventions was conducted based on adverse events reported in the trials. Seven studies had no adverse events during the process. Seven studies reported adverse effects during treatment with iTBS, rTMS, tDCS, NMES, CTAR, shaker and acupuncture. Specifically, iTBS and rTMS were linked to tolerable dizziness, while tDCS caused mild itching. NMES resulted in skin irritation at the electrodes, and CTAR and Shaker exercises led to transient pain in some participants. Adverse effects associated with acupuncture included discomfort, hematoma and pain. The remaining studies did not report on adverse events.

### 3.10 Publication Bias and Sensitivity Analysis

Egger's test showed no significant small‐study bias in any of the comparisons for swallowing function, and feeding and daily functioning (*p* = 0.241 and 0.961, respectively). (Supporting Information S4).

Sensitivity analysis was performed on the results for both swallowing function and feeding and daily function. There was no significant change in the results, further illustrating the stability of the study results (Supporting Information S4).

## 4 Discussion

To our knowledge, this is the first network meta‐analysis investigating the effects of different interventions on swallowing function in patients with post‐stroke dysphagia. In this network meta‐analysis, we included evidence from 33 RCTs involving 1,341 participants. We found that acupuncture was the most effective intervention for improving patients' swallowing function, and feeding and daily function, followed by CTAR.

Previously paired meta‐analyses have demonstrated that all three interventions, namely the peripheral and central stimulation methods, the muscle strength exercise and the traditional Chinese medicine intervention, were effective in improving swallowing function in patients with stroke swallowing disorders. This finding is consistent with the results of our study [36, 37]. From a more comprehensive clinical perspective, our network meta‐analysis provides important insights into swallowing rehabilitation, highlighting which interventions are more effective not only for improving swallowing ability but also for enhancing feeding daily function in post‐stroke dysphagia patients.

Swallowing involves a rapid, highly coordinated set of neuromuscular actions, from lip closure to upper oesophageal sphincter closure [38]. The central coordination of this complex sensorimotor task relies on an extensive network of cortical, subcortical and brainstem structures [39]. Stroke is the most common disorder that disrupts this swallowing network, resulting in post‐stroke dysphagia [38].

### 4.1 Traditional Chinese Medicine

Acupuncture is the most effective intervention when all three types are compared together. Possible reasons for its effectiveness include improving blood circulation through acupoints stimulation, stimulating nerve motor fibres, repairing damaged medullary arc function after stroke, accelerating neural plasticity reorganisation, stimulating swallowing muscle contraction, increasing muscle strength and facilitaing dysphagia recovery [40]. The European Stroke Organisation and European Society for Swallowing Disorders guidelines also recommend acupuncture for the treatment of post‐stroke dysphagia treatment [38]. However, acupuncture requires specialised and trained practitioners of traditional Chinese medicine, which can lead to higher treatment costs and lower economic benefits.

### 4.2 Swallowing Exercises Focusing on Muscle Strength

The results of the study found that CTAR and shaker ranked second and third, respectively, in improving swallowing function in stroke patients. In addition, CTAR ranked fourth in improving feeding and daily function. CTAR facilitates swallowing rehabilitation by placing an elastic rubber ball under the patient's chin, which activates isotonic and isometric muscle contractions of the supraglenoid muscle through chin tucking on the rubber ball, ultimately strengthening the supraglenoid muscle [41]. The shaker exercise allows patients to look up at their toes while in the supine position, promoting isometric and isotonic contraction movements of the muscles involved in swallowing [42]. Both CTAR and shaker exercises aim to restore swallowing function by strengthening swallowing‐related muscles. According to the study of Park et al. [37], CTAR not only activates the suprahyoid muscle more than shaker exercises but also selectively activates the sternocleidomastoid muscles to a greater extent, thereby enhancing the patients' exercise compliance. Furthermore, Park et al. [43] used gamification for CTAR training and found that it not only effectively improved patients' swallowing function, but also their compliance and interest in exercises compared to the conventional method. With the rapid development of video games, virtual reality and artificial intelligence, future research could explore integrating these new technologies with muscle‐strengthening exercises to achieve intelligent rehabilitation.

### 4.3 Peripheral and Central Stimulation Methods

Chiang et al. [44] conducted a network analysis of noninvasive neurostimulation therapies. Similar to their findings, we concluded that the difference in swallowing function improvement between PES and sham groups was not significant. PES increases pharyngeal motor cortical excitability through pulse stimulation and factors such as potential under‐treatment and the inclusion of patients with mild dysphagia, who may experience spontaneous recovery of swallowing function, could have contributed to the neutral results of the trial [45]. Additionally, PES requires the insertion of an endoluminal catheter via the nose or mouth, which may cause discomfort for patients [46]. In contrast to the study by Chiang et al. [44], our results showed that rTMS was the most effective neurostimulation therapy, with NMES following as the second most effective. We considered that NMES was more effective as it targets the peripheral neuromuscular system to strengthen weakened oropharyngeal muscle tissue, while rTMS stimulates the pharyngeal motor cortex to promote neuroplasticity after a stroke [47]. NMES is also the most cost‐effective and easiest method to apply among electrical stimulation methods [47]. The differences in results between our study and that of Chiang et al. may stem from their inclusion of only comparative studies involving NMES combined with conventional rehabilitation, which limited their ability to assess the single effect of NMES. In contrast, we included a study that compared NMES with a placebo [48], further clarifying its effectiveness.

### 4.4 Safety

According to our study, some adverse effects, such as pain, were reported for all three types of interventions. Still, these adverse effects were all transient and no participant refused training due to them [26, 27]. This indicated that all three interventions were safe and effective. However, 57.6% of the included studies did not report adverse effects, indicating that the safety of these interventions should be further elucidated in future studies.

### 4.5 Practice Implications

Post‐stroke dysphagia can lead to serious complications such as aspiration pneumonia, malnutrition and dehydration, often requiring additional respiratory care and enteral diet care [49]. Therefore, it is important for medical staff to acquire scientific knowledge and skills related to post‐stroke dysphagia rehabilitation. The study indicated that timely interventions in rehabilitation management, nutritional care, prevention of possible complications, patient assessment and treatment education for dysphagia could reduce the incidence of patient aspiration [50]. The results of this study can provide information on rehabilitation training options for dysphagia and promote the effective application of rehabilitation interventions.

### 4.6 Limitations

Our study has several strengths in providing evidence for frontline healthcare professionals by comparing established swallowing rehabilitation interventions. This study strictly followed PRISMA guidelines and included only RCTs, which lends greater rigour and reliability to our findings. However, there are also some limitations. Firstly, the duration of interventions varied, with shorter durations for stimulation methods and longer durations for muscle strength exercises. There was no further analysis conducted on the impact of different intervention durations or the long‐term benefits. Secondly, the included studies used different swallowing function assessment scales and the results indicators analysed used SMD. Thus, the results need to be interpreted with caution. Finally, the quality analyses of three of the included studies analyses were rated as low. Although our investigation had no publication bias or inconsistency, future studies need to prioritise the implementation of blinding and allocation concealment to reduce selection bias and enhance the reliability of the study results.

## 5 Conclusions

This network meta‐analysis indicated that acupuncture, CTAR and shaker may be the most effective interventions to improve swallowing function in stroke patients. Another key finding of this study was that acupuncture, iTBS, tDCS and CTAR were probably the best interventions to improve feeding and daily function. Considering that CTAR is a low‐cost and highly feasible intervention that can be mastered by medical staff, we recommend its selection as a rehabilitation measure for patients with post‐stroke dysphagia. This can help improve their swallowing function, reduce the incidence of aspiration and aspiration pneumonia and improve patients' quality of life. Future studies are encouraged to further explore this field and design high‐quality RCTs, emphasising strict control of allocation concealment and subject blinding. Understanding the various interventions is crucial for medical staff to implement care correctly and improve patient satisfaction and participation.

## Author Contributions

**Bohan Zhang:** conceptualisation, methodology, software, formal analysis, investigation, writing‐original draft, visualisation. **Ka Po Wong:** conceptualisation, methodology, writing – review & editing, visualisation. **Cai Guo:** investigation, validation. **Shu‐Cheng Chen:** investigation, validation. **Shuojin Fu:** investigation. **Ruifu Kang:** investigation. **Qian Xiao:** validation, writing – review & editing. **Jing Qin:** resources, writing – review & editing, supervision.

## Ethics Statement

The protocol for this study was registered with PROSPERO (registration number: CRD42023391951).

## Conflicts of Interest

The authors declare no conflicts of interest.

### Peer Review

The peer review history for this article is available at https://www.webofscience.com/api/gateway/wos/peer‐review/10.1111/joor.13901.

## Supporting information


Supporting Information S1.

Supporting Information S2.

Supporting Information S3.

Supporting Information S4.

## Data Availability

Data supporting the results of this study can be obtained from the corresponding author upon reasonable request.
