# Supplementary material for: Effects of Non‐Pharmacological Interventions on the Swallowing Function of Patients With Post‐Stroke Dysphagia: A Systematic Review and Network Meta‐Analysis
Source: J Oral Rehabil. 2024 Nov 12;52(1):109–20. doi: 10.1111/joor.13901 (PMC11680505; doi:10.1111/joor.13901)
Supplement: Supplementary file 2 — Supporting Information S2. [file JOOR-52-109-s004.docx]

The detailed Pubmed search strategy

#1. [mh ^"cerebrovascular disorders"] or [mh "basal ganglia cerebrovascular disease"] or [mh "brain ischemia"] or [mh "carotid artery diseases"] or [mh "intracranial arterial diseases"] or [mh “intracranial arteriovenous malformations”] or [mh "intracranial embolism and thrombosis"] or [mh "intracranial hemorrhages"] or [mh ^stroke] or [mh "brain infarction"]

#2. [mh ^"brain injuries"] or [mh ^"brain injury, chronic"]

#3. (stroke or cva or poststroke or "post-stroke" or cerebrovasc* or cerebral next vasc*):ti,ab

#4.((cerebral* or cerebell* or brain* or vertebrobasilar) near/5 (isch*emi* or infarct* or thrombo* or emboli* or apoplexy*)):ti,ab

#5. ((brain* or cerebral* or subarachnoid) near/5 (haemorrhage* or hemorrhage* or haematoma* or hematoma* or bleed*)):ti,ab

#6. [mh ^hemiplegia] or [mh paresis]

#7. (hemipleg* or hemipar* or paresis or paretic or brain next injur*):ti,ab

#8. [mh ^"neurologic"]

#9. #1 or #2 or #3 or #4 or #5 or #6 or #7 or #8

#10. [mh ^” deglutition disorders”]

#11. (“swallowing disorder” or dysphagia or “oropharyngeal dysphagia” or “esophageal dysphagia”):ti,ab

#12. (dysphag*):ti,ab

#13. #10 or #11 or #12

#14. #9 and #13
