# Supplementary material for: Effects of Non‐Pharmacological Interventions on the Swallowing Function of Patients With Post‐Stroke Dysphagia: A Systematic Review and Network Meta‐Analysis
Source: J Oral Rehabil. 2024 Nov 12;52(1):109–20. doi: 10.1111/joor.13901 (PMC11680505; doi:10.1111/joor.13901)
Supplement: Supplementary file 3 — Supporting Information S3. [file JOOR-52-109-s002.docx]

The study characteristics of the included studies (n=33).

| Study  Authors, year | Country | Intervention | Sample | Sex  M/F | Age, years (mean±SD) | Stroke Type  ischemic/ hemorrhagic | Stroke onset  (mean±SD) | Implementation details (process, dosage or duration) | Adverse reported | Outcome |
| --- | --- | --- | --- | --- | --- | --- | --- | --- | --- | --- |
| Xie Yu-Lei 2022 ^1^ | China | iTBS  rTMS | 24  23 | 16/8  18/5 | 67.5±10.6  64.8±11.3 | 6/18  7/16 | 25.1±11.74d  29.9±17.11d | iTBS: 100% of RMT, 3 pulses of 50 Hz bursts repeated at 5 Hz (2s on and 8s off) for a total of 192 seconds (600 pulses) on the “hot spot” of affected hemisphere  rTMS: 10 Hz and 100% RMT with 2s trains at an intertrain interval of 18 seconds for 20 minutes (1200 pulses) on the same position  10days | No adverse events occurred | WST |
| Tingwei Wang 2022 ^2^ | China | TPRT  CDT | 18  18 | 10/8  11/7 | 60.2±8.8  60.7±8.6 | 10/8  9/9 | 4.9±1.9m  4.7±1.8m | TPRT: Squeeze the device hard upward with tongue  CDT: oral-facial motor control exercises, oral-facial muscles sensory stimulation  20 min/day, 5 days a week for 4 weeks | No adverse events occurred | PAS |
| Elif Tarihci Cakmak 2022 ^3^ | Turkey | NMES  CDT | 17  17 | 11/6  9/8 | 62.9±9.8  63.6±10.0 | 16/1  15/2 | 48.3±92.6w  52.2±92.2w | NMES: 80 Hz pulse rate with a biphasic pulse duration of 700 μs and stimulation intensity not exceeding 25 mA  CDT: diet modification according to the patients’ oral intake levels, oral hygiene education, compensatory methods, and exercises  45 min/day, 5 days a week for 3 weeks | NR | EAT-10, SWAL-QOL |
| Jinzhu Rao 2022 ^4^ | China | iTBS  sham iTBS | 33  31 | 22/11  24/7 | 63.4±10.4  65.9±11.4 | 15/18  19/12 | 22.0±11.1d  26.0±11.9d | iTBS: 3 pulses of stimulation delivered at 50 Hz and repeated at 5 Hz, in which a 2 s train of TBS was repeated every 10 s for a total of 190 s (600 pulses in total). The stimulation intensity was 100% RMT  sham iTBS: the probe was placed perpendicular to the patient’s skull so that the signal could not pass through the skull to act on the brain  5 days a week for 2 weeks | Three participants in the iTBS group had slight dizziness, which was tolerable, and the experiment could be continued | PAS |
| Huiyu Liu 2022 ^5^ | China | rTMS  sham rTMS | 23  26 | 17/6  20/6 | 67.6±11.7  67.7±10.0 | 19/4  21/5 | 74.2±88.2d  63.4±59.5 | rTMS: a 5-Hz rTMS protocol was applied to the affected mylohyoid cortical region of the affected hemisphere at an intensity of 80% of RMT, a stimulation time of 2 s, and an interval of 10 s between each stimulation  sham rTMS: the coil is held at 90◦ to the scalp during the intervention  20 min/day, 5 days a week for 2 weeks | Two patients in the rTMS group reported dizziness after several rTMS interventions | PAS |
| Sandeep Kumar 2022 ^6^ | USA | tDCS  sham tDCS | 14  15 | 3/11  6/9 | 68.0±12.6  73.0±14.1 | NR | NR | tDCS: 2 mA tDCS twice daily for a total of 20 min  sham tDCS: sham stimulation  20 min/day, twice daily, 5 days | No adverse events occurred | PAS |
| Sima Farpour 2022 ^7^ | Iran | tDCS  sham tDCS | 22  22 | 13/9  10/12 | 65.3±16.3  70.7±16.3 | NR | 4.1±4.0d  4.5±4.0d | tDCS: 2 mA for 20 min for five days (one session in a day). The current density of the tDCS anodal pad used was 0.125 mA/cm^2^  sham tDCS: the current only for 30s at the beginning and the end of the period  20 min/day, 5 days | Itching (which was tolerable) was reported for 3 patients in the tDCS group | MASA, FOIS |
| Ayse Güleç 2021 ^8^ | Turkey | NMES  CDT | 12  13 | 7/5  12/1 | 67.0±4.6  63.3±6.3 | 11/1  13/0 | 7.0±8.5m  4±10.5m | NMES: Electrical stimulation was delivered with pulsed current at a fixed pulse rate of 80 Hz and a pulse duration of 700 μs  CDT: oral exercises, thermal–tactile stimulation, compensatory techniques, and swallowing therapeutic maneuvers  30 min/day, 3 days a week for 5 weeks | NR | PAS, FOIS |
| Viridiana Arreola 2021 ^9^ | Spain | NMES  CDT | 29  29 | 19/10  19/10 | 70.7±12.9  73.5±11.6 | 28/1  28/1 | 466.3±903.5d  630.4±1247.8d | NMES: the current intensity was set to 100% of the motor threshold  CDT: adaptation of fluids with thickening agents, texture-modified diet, oral hygiene recommendations, postural changes if necessary, and nutritional advice  two 1-hour sessions per day the first week and one 1hour session per day the second week (15 sessions) | One patient had skin irritation in the electrode zone | PAS |
| Ji-Su Park 2020 ^10^ | Korea | CTAR  sham CTAR | 15  14 | 9/6  8/6 | 62.1±10.1  61.8±12.1 | 7/8  8/6 | NR | CTAR: isometric and isotonic exercises  sham CTAR: using a within 1-mmthick device with almost no resistance to the suprahyoid muscles  30 min/day, 5 days a week for 4 weeks | No adverse events occurred | FOIS |
| Mei-Yun Liaw 2020 ^11^ | China | EMST  CDT | 10  11 | 3/7  9/2 | 61.2±10.7  66.8±11.5 | 6/4  3/8 | 3.0±2.0m  2.4±0.7m | EMST: using the Dofin Breathing Trainer, patients were instructed to exhale slowly and gently through the mouthpiece  CDT: postural training, breathing control, improving cough technique, checking chest wall mobility, fatigue management, orofacial exercises  5 days a week for 6 weeks | NR | MRS, BI |
| Maryam Tarameshlu 2019 ^12^ | Iran | rTMS  CDT | 6  6 | 4/2  1/5 | 55.3±19.6  74.7±5.9 | NR | 3.2±2.1m  5.3±3.4m | rTMS: the intact cerebral hemisphere with a train of 1200 pulses at 1 Hz, with stimulus strength at 20% above the resting motor threshold for 20 min, 5 daily consecutive days  CDT: postural changes, alerting volume and speed of food presentation, alerting food consistency and viscosity, and improving sensory oral awareness | NR | FOIS |
| Marilia Simonelli 2019 ^13^ | Italy | NMES  CDT | 17  16 | 11/6  6/10 | 67.2±16.2  72.4±12.3 | NR | 45.2±22.3d  32.6±18.1d | NMES: The typical level electrical stimulus, at 80 Hz and at 300 microsec, ranged from min 7.8 to max 12.5 mA with a mean level of 9.3 mA  CDT: oral-facial, lingual, and laryngeal motor exercises  Two 30 min/day, 5 days a week for 8 weeks | No adverse events occurred | PAS, FOIS |
| Ji-Su Park 2019 ^14^ | Korea | CTAR  Shaker | 19  18 | 12/7  9/9 | 61.0±11.2  59.5±9.3 | 8/11  5/13 | 3.6±1.2m  3.9±1.2 | CTAR: using the LES 100 device, isometric and isotonic exercises in combination with the game  Shaker: isometric and isotonic exercises  5 days a week for 4 weeks | 4 patients reported temporary pain, fatigue and discomfort in the neck | PAS, FOIS |
| Hee-Su Park 2019 ^15^ | Korea | TPRT  CDT | 12  12 | 6/6  5/7 | 66.5±9.5  64.8±11.2 | NR | 24.4±8.7w  25.7±6.3w | TPRT: Patients were asked to push the tongue firmly onto the palate, while squeezing neck muscles, and swallow as forcefully as possible  CDT: swallow naturally without intentional force from the tongue and neck muscles  5 days a week for 4 weeks | NR | VDS |
| Hwan‐Hee Kim 2019 ^16^ | Korea | CTAR  CDT | 12  13 | 6/6  6/7 | 63.5±5.5  65.2±6.2 | 7/5  6/7 | NR | CTAR: isotonic and isometric exercise  CDT: oral facial massage, thermal‐tactile stimulation and various compensatory trainings  5 days a week for 6 weeks | NR | PAS |
| Sonja Suntrup-Krueger 2018 ^17^ | Germany | tDCS  sham tDCS | 29  30 | 17/12  17/13 | 68.9±11.5  67.2±14.5 | 29/0  30/0 | 116.3±98.9h  116.8±64.9h | tDCS: 1mA for 20 minutes, once daily on 4 consecutive days  sham tDCS: stimulation lasted for only 30 seconds with the electrodes left in place for a further 20 minutes | NR | FEDSS |
| Lise Sproson 2018 ^18^ | UK | NMES  CDT | 13  14 | 8/5  8/6 | 76.0±11.4  79.0±11.4 | NR | 17.3±25.0m  9.1±20.5m | NMES: A pulse rate of 30 Hz was used, during each 5-s pulse of stimulation, participants were instructed to undertake a repetition of the exercise for that 10-min section  CDT: posture and diet modification  30 min/day, 5 days a week for 4 weeks | NR | SWAL-QOL, DSRS |
| Ji-Su Park 2018 ^19^ | Korea | CTAR  CDT | 11  11 | 6/5  4/7 | 62.2±17.3  58.4±12.5 | 7/4  6/5 | 27.2±8.5w  32.1±14.4w | CTAR: In isometric CTAR, the patients are asked to chin tuck against device 3 times for 60 s with no repetition. In isotonic CTAR, the patient performs 30 consecutive repetitions by strongly pressing against the resistance of the device and releasing it again  CDT: orofacial muscle exercises, thermal tactile stimulation, and therapeutic or compensatory maneuvers  30 min/day, 5 days a week for 4 weeks | NR | PAS |
| Jong-Hoon Moona 2018 ^20^ | Korea | TPRT  CDT | 8  8 | 3/5  4/4 | 62.0±4.2  63.5±6.1 | 6/2  6/2 | 56.0±17.4d  59.9±20.0d | TPRT: an anterior and posterior isometric tongue strength exercise and an isometric tongue accuracy exercise  CDT: thermal tactile stimulation, the Mendelsohn maneuver, and diet modification  30 min/day, 5 days a week for 8 weeks | NR | MASA, SWAL-QOL |
| Young Hyun Ahn 2017 ^21^ | Korea | tDCS  sham tDCS | 13  13 | 9/4  6/7 | 61.6±10.3  66.4±10.7 | 5/8  11/2 | 12.3±4.9m  11.62±4.6m | tDCS: a total of 10 sessions of 20 min and 1mA stimulation (5 times per week for 2 weeks)  sham tDCS: 1 mA current was delivered for only 30 s through 2 anodal electrodes, producing an initial tingling sensation but no significant changes in cortical excitability | NR | DOSS |
| Jong Hoon Moon 2017 ^22^ | Korea | EMST  CDT | 9  9 | 6/3  6/3 | 63.0±5.8  63.1±5.2 | 6/3  7/2 | 21.4±5.1d  21.1±4.0d | EMST: taking a deep breath and biting a mouthpiece, during which time the patient was told to blow faster and stronger  CDT: orofacial exercises, thermal-tactile stimulation, the Mendelson maneuver  30 min/day, 5 days a week for 4 weeks | NR | PAS |
| Mi-Ja Eom 2017 ^23^ | Korea | EMST  sham EMST | 13  13 | 5/8  6/7 | 69.2±4.1  70.2±3.6 | NR | NR | EMST: Patients were asked to hold a Expiratory Muscle Trainer pressure in their mouth in the state of maximum inhalation and exhale strongly and rapidly  sham EMST: using a sham EMST device with no loading device  5 days a week for 4 weeks | NR | PAS |
| H. D. KIM 2017 ^24^ | Korea | TPRT  CDT | 18  17 | 11/7  8/9 | 62.2±11.0  59.3±10.2 | 11/7  10/7 | 4.9±5.5m  5.3±5.6m | TPRT: pressing the tongue strongly against the palate  CDT: thermal tactile stimulation, facial massage and various manoeuvres  5 days a week for 4 weeks | NR | PAS |
| Jing Gao 2017 ^25^ | China | CTAR  Shaker  CDT | 30  30  30 | 13/17  15/15  14/16 | 70.9±6.6  71.1±7.1  71.1±6.4 | NR | NR | CTAR: patients seated while tucking the chin to compress an inflatable rubber ball as far as possible  Shaker: patients were in supine position, and then raised their head and neck to look at their feet, which was a single action  CDT: tongue exercises and mouth exercises  7 days a week for 6 weeks | NR | VFSS |
| Jong-Bae Choi 2017 ^26^ | Korea | Shaker  CDT | 16  15 | 10/6  9/6 | 60.8±10.9  60.4±10.5 | 9/7  12/3 | 3.4±11.2  4.13±1.0 | Shaker: participants sustained 3 head lifts held for 60 s without movement in the supine position; a 60-s rest was allowed between lifts  CDT: orofacial muscle exercises, thermal tactile stimulation, and therapeutic or compensatory maneuvers  30 min/day, 5 days a week for 4 weeks | 5 patients reported temporary fatigue and pain | PAS |
| Wenguang Xia 2016 ^27^ | China | Acupuncture  CDT | 60  60 | 34/26  35/25 | 65.3±14.2  66.1±14.3 | 43/17  41/19 | 9.3±2.3d  8.7±2.5d | Acupuncture: The nape, scalp, and tongue acupuncture  CDT: Functional training was applied to the feeding and swallowing organs  30 min/day, 6 days a week for 4 weeks | discomfort (12 patients), hematoma (two patients), and severe pain (one patient) | DOSS |
| J.-S. Park 2016 ^28^ | Korea | NMES  sham NMES | 25  25 | 12/13  14/11 | 54.0±11.9  55.8±12.2 | NR | 35.4±5.6w  36.0±6.1w | NMES: The intensity was increased gradually at an interval of 0.5 mA  sham NME: the minimum stimulation that was needed for the patient to feel a tingling sensation was applied  30 min/day, 6 days a week for 6 weeks | NR | VDS |
| J.-S. Park 2016 ^29^ | Korea | EMST  sham EMST | 14  13 | 6/8  6/7 | 64.3±10.7  65.8±11.3 | NR | 27.4±6.3w  26.6±6.8w | EMST: subjects were asked to open their mouth following maximum inhalation and locate the EMST mouthpiece between the lips before closing their mouth. Then, they were instructed to blow strong and fast until the pressure release valve within the EMST device opens.  sham EMST: using a sham device with no spring loading within the device  5 days a week for 4 weeks | NR | FOIS |
| Philip M. Bath 2016 ^30^ | UK | PES  sham PES | 70  56 | 48/22  46/20 | 74.0±9.9  74.9±12.6 | 61/9  48/8 | 12.6±9.5d  14.4±10.0d | PES: the catheter was connected to the controlling base station, and electric current at 5 Hz was increased incrementally from 1 mA to detect threshold and then tolerated intensity levels in all patients  sham PES: no stimulation after establishment of threshold and tolerated levels  Three days | No adverse events occurred | PAS, DSRS |
| Kyoung Don Kim 2015 ^31^ | Korea | CTAR  Shaker | 13  13 | 8/5  7/8 | 63.2±10.2  63.6±8.1 | NR | 15.6±2.9m  16.2±3.1m | CTAR: the short neck flexion exercises  Shaker: For the isometric Shaker exercise, patients lay on the bed and raised their heads without moving their shoulders off the bed, looked at the ends of their feet for 60 seconds, and then lowered their heads back on the bed and rested for 60 seconds; the isotonic Shaker exercise had the patients raise their heads in the same posture and look at the ends of their feet 30 consecutive times.  30 min/day, 3 days a week for 6 weeks | NR | VFSS |
| Kyeong Woo Lee 2014 ^32^ | Korea | NMES  CDT | 31  26 | 22/9  20/6 | 63.4±11.4  66.7±9.5 | NR | 5.0±1.4d  5.5±1.1d | NMES: stimulation intensity was set at 120% of the mean threshold value. A pulse rate of 80 Hz was used with 700 μs duration  CDT: thermal-tactile stimulation with any combination of lingual-strengthening exercises, laryngeal adduction-elevation exercises  30 min/day, 5 days a week for 3 weeks | No adverse events occurred | FOIS |
| J.-W. Park 2013 ^33^ | Korea | rTMS  sham rTMS | 9  9 | 5/4  5/4 | 73.7±3.8  68.9±9.3 | 7/2  8/1 | 59.9±16.3d  63.9±26.8d | rTMS: A session of stimulation consisted of 10 trains of 5-Hz stimulation, each lasting for 10 s and then repeated every minute given through a 70 mm figure-of-eight coil positioned over the pharyngeal hot spot of the intact hemisphere  sham rTMS: using a 90% coil tilt, which produces the same noise as active stimulation, but has been shown not to produce motor cortical stimulation  10 min/day, 5 days a week for 2 weeks | NR | VDS |

Abbreviations: iTBS: Intermittent theta burst stimulation; rTMS: Repetitive transcranial magnetic stimulation; RMT: Resting motor threshold; WST: Water-swallowing test; NR: Not report; TPRT: Tongue-pressure resistance training; CDT: Conventional dysphagia training; PAS: Penetration Aspiration Scale; NMES: Neuromuscular electrical stimulation; SWAL-QOL: The swallowing quality of life questionnaire; tDCS: Transcranial direct current stimulation; FOIS: The Functional Oral Intake Scale; MASA: The Mann Assessment of Swallowing Ability; EAT-10: The eating assessment tool; CTAR: Chin Tuck against resistance exercise; MRS: Modified Rankin Scale; BI: Barthel index; VDS: The Videofluoroscopic Dysphagia Scale; FEDSS: Fiberoptic Endoscopic Dysphagia Severity Scale; DOSS: Dysphagia Outcome and Severity Scale; EMST: Expiratory muscle strength training; VFSS: Video fluoroscopic swallowing study; DSRS: the clinical Dysphagia Severity Rating Scale; PES: Pharyngeal electric stimulation

# References

1. Yu-Lei X, Shan W, Ju Y, et al: Theta burst stimulation versus high-frequency repetitive transcranial magnetic stimulation for poststroke dysphagia: A randomized, double-blind, controlled trial. *Medicine* 2022; 101: e28576

2. Wang T, Tai J, Hu R, et al: Effect of Tongue-Pressure Resistance Training in Poststroke Dysphagia Patients with Oral Motor Dysfunction-A Randomized Controlled Trial. *Am J Phys Med Rehabil* 2022; 101(12):1134-1138

3. Tarihci Cakmak E, Sen EI, Doruk C, et al: The Effects of Neuromuscular Electrical Stimulation on Swallowing Functions in Post-stroke Dysphagia: A Randomized Controlled Trial. *Dysphagia* 2022

4. Rao J, Li F, Zhong L, et al: Bilateral Cerebellar Intermittent Theta Burst Stimulation Combined With Swallowing Speech Therapy for Dysphagia After Stroke: A Randomized, Double-Blind, Sham-Controlled, Clinical Trial. *Neurorehabil Neural Repair* 2022; 36(7): 437-448

5. Liu H, Peng Y, Liu Z, et al: Hemodynamic signal changes and swallowing improvement of repetitive transcranial magnetic stimulation on stroke patients with dysphagia: A randomized controlled study. *Front Neurol* 2022; 13: 918974

6. Kumar S, Marchina S, Langmore S, et al: Fostering eating after stroke (FEASt) trial for improving post-stroke dysphagia with non-invasive brain stimulation. *Sci Rep* 2022; 12(1):9607

7. Farpour S, Asadi-Shekaari M, Borhani Haghighi A, et al: Improving Swallowing Function and Ability in Post Stroke Dysphagia: A Randomized Clinical Trial. *Dysphagia* 2022: 1-10

8. Güleç A, Albayrak I, Erdur Ö, et al: Effect of swallowing rehabilitation using traditional therapy, kinesiology taping and neuromuscular electrical stimulation on dysphagia in post-stroke patients: A randomized clinical trial. *Clin Neurol Neurosurg* 2021; 211: 107020

9. Arreola V, Ortega O, Álvarez-Berdugo D, et al: Effect of Transcutaneous Electrical Stimulation in Chronic Poststroke Patients with Oropharyngeal Dysphagia: 1-Year Results of a Randomized Controlled Trial. *Neurorehabil Neural Repair* 2021; 35: 778-789

10. Park JS, An DH, Kam KY, et al: Effects of resistive jaw opening exercise in stroke patients with dysphagia: A double- blind, randomized controlled study. *J Back Musculoskelet Rehabil* 2020; 33(3): 507-513

11. Liaw MY, Hsu CH, Leong CP, et al: Respiratory muscle training in stroke patients with respiratory muscle weakness, dysphagia, and dysarthria - a prospective randomized trial. *Medicine* 2020; 99: e19337

12. Tarameshlu M, Ansari NN, Ghelichi L, et al: The effect of repetitive transcranial magnetic stimulation combined with traditional dysphagia therapy on poststroke dysphagia: a pilot double-blinded randomized-controlled trial. *Int J Rehabil Res.* 2019; 42(2): 133-138

13. Simonelli M, Ruoppolo G, Iosa M, et al: A stimulus for eating. The use of neuromuscular transcutaneous electrical stimulation in patients affected by severe dysphagia after subacute stroke: A pilot randomized controlled trial. *NeuroRehabilitation* 2019; 44: 103-110

14. Ji-Su P, Gihyoun LEE, Young-Jin J: Effects of game-based chin tuck against resistance exercise vs head-lift exercise in patients with dysphagia after stroke: An assessor-blind, randomized controlled trial. *J Rehabil Med* 2019; 51: 749‐754

15. Park HS, Oh DH, Yoon T, et al: Effect of effortful swallowing training on tongue strength and oropharyngeal swallowing function in stroke patients with dysphagia: a double-blind, randomized controlled trial. *Int J Lang Commun Disord* 2019; 54(3): 479-484

16. Kim HH, Park JS: Efficacy of modified chin tuck against resistance exercise using hand-free device for dysphagia in stroke survivors: A randomised controlled trial. *J Oral Rehabil* 2019; 46(11): 1042-1046

17. Suntrup-Krueger S, Ringmaier C, Muhle P, et al: Randomized trial of transcranial direct current stimulation for poststroke dysphagia. *Ann Neurol* 2018; 83: 328‐340

18. Sproson L, Pownall S, Enderby P, et al: Combined electrical stimulation and exercise for swallow rehabilitation post-stroke: a pilot randomized control trial. *Int J Lang Commun Disord* 2018; 53: 405-417

19. Park JS, An DH, Oh DH, et al: Effect of chin tuck against resistance exercise on patients with dysphagia following stroke: A randomized pilot study. *NeuroRehabilitation* 2018; 42: 191-197

20. Moon JH, Hahm SC, Won YS, et al: The effects of tongue pressure strength and accuracy training on tongue pressure strength, swallowing function, and quality of life in subacute stroke patients with dysphagia: a preliminary randomized clinical trial. *Int J Rehabil Res* 2018; 41: 204-210

21. Young Hyun AHN, Hyun-Joo S, Jin-Sung P, et al: Effect of bihemispheric anodal transcranial direct current stimulation for dysphagia in chronic stroke patients: A randomized clinical trial. *J Rehabil Med* 2017; 49(1): 30‐35

22. Moon JH, Jung J, Won YS, et al. Effects of expiratory muscle strength training on swallowing function in acute stroke patients with dysphagia. *Journal of Physical Therapy Science* 2017; 29: 609‐612.

23. Mi-Ja E, Moon-Young C, Dong-Hwan O, et al: Effects of resistance expiratory muscle strength training in elderly patients with dysphagic stroke. *NeuroRehabilitation* 2017; 41: 747‐752

24. Kim HD, Choi JB, Yoo SJ, et al: Tongue-to-palate resistance training improves tongue strength and oropharyngeal swallowing function in subacute stroke survivors with dysphagia. *J Oral Rehabil* 2017; 44: 59-64

25. Gao J, Zhang HJ: Effects of chin tuck against resistance exercise versus Shaker exercise on dysphagia and psychological state after cerebral infarction. *Eur J Phys Rehabil Med* 2017; 53: 426-432

26. Jong-Bae C, Sun-Hwa S, Jong-Eun Y, et al: Effects of Shaker exercise in stroke survivors with oropharyngeal dysphagia. *NeuroRehabilitation* 2017; 41(4): 753‐757

27. Xia W, Zheng C, Zhu S, et al: Does the addition of specific acupuncture to standard swallowing training improve outcomes in patients with dysphagia after stroke? a randomized controlled trial. *Clin Rehabil* 2016; 30: 237-246

28. Park JS, Oh DH, Hwang NK, et al: Effects of neuromuscular electrical stimulation combined with effortful swallowing on post-stroke oropharyngeal dysphagia: a randomised controlled trial. *J Oral Rehabil* 2016; 43: 426-434

29. Park JS, Oh DH, Chang MY, et al: Effects of expiratory muscle strength training on oropharyngeal dysphagia in subacute stroke patients: a randomised controlled trial. *J Oral Rehabil* 2016; 43: 364-372

30. Bath PM, Scutt P, Love J, et al: Pharyngeal Electrical Stimulation for Treatment of Dysphagia in Subacute Stroke: A Randomized Controlled Trial. *Stroke* 2016; 47: 1562-1570

31. Don Kim K, Lee HJ, Lee MH, et al: Effects of neck exercises on swallowing function of patients with stroke. *J Phys Ther Sci* 2015; 27: 1005-1008

32. Lee KW, Kim SB, Lee JH, et al: The effect of early neuromuscular electrical stimulation therapy in acute/subacute ischemic stroke patients with Dysphagia. *Ann Rehabil Med* 2014; 38: 153-159

33. Park JW, Oh JC, Lee JW, et al: The effect of 5Hz high-frequency rTMS over contralesional pharyngeal motor cortex in post-stroke oropharyngeal dysphagia: a randomized controlled study. *Neurogastroenterol Motil* 2013; 25: 324-e250
