# Supplementary material for: Effects of Non‐Pharmacological Interventions on the Swallowing Function of Patients With Post‐Stroke Dysphagia: A Systematic Review and Network Meta‐Analysis
Source: J Oral Rehabil. 2024 Nov 12;52(1):109–20. doi: 10.1111/joor.13901 (PMC11680505; doi:10.1111/joor.13901)

1.The risk of bias

2. The inconsistency test


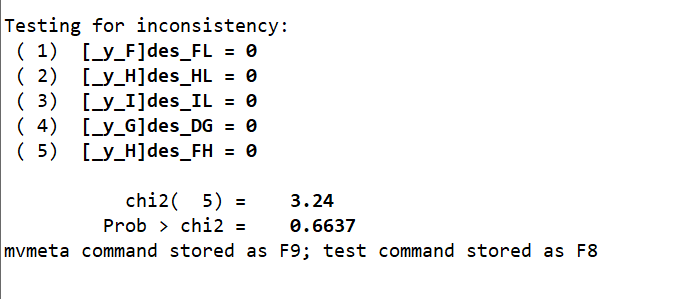


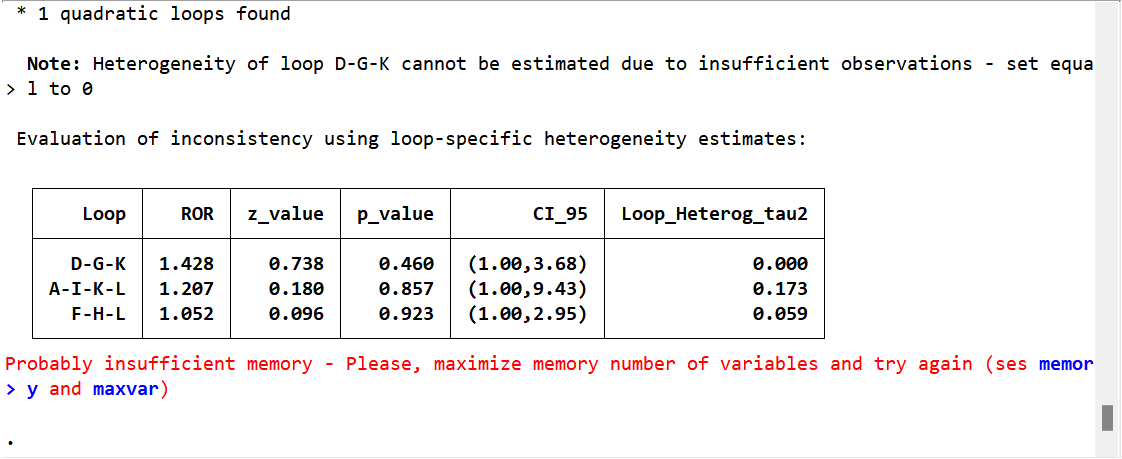


3. Paired meta-analysis forest of swallowing function


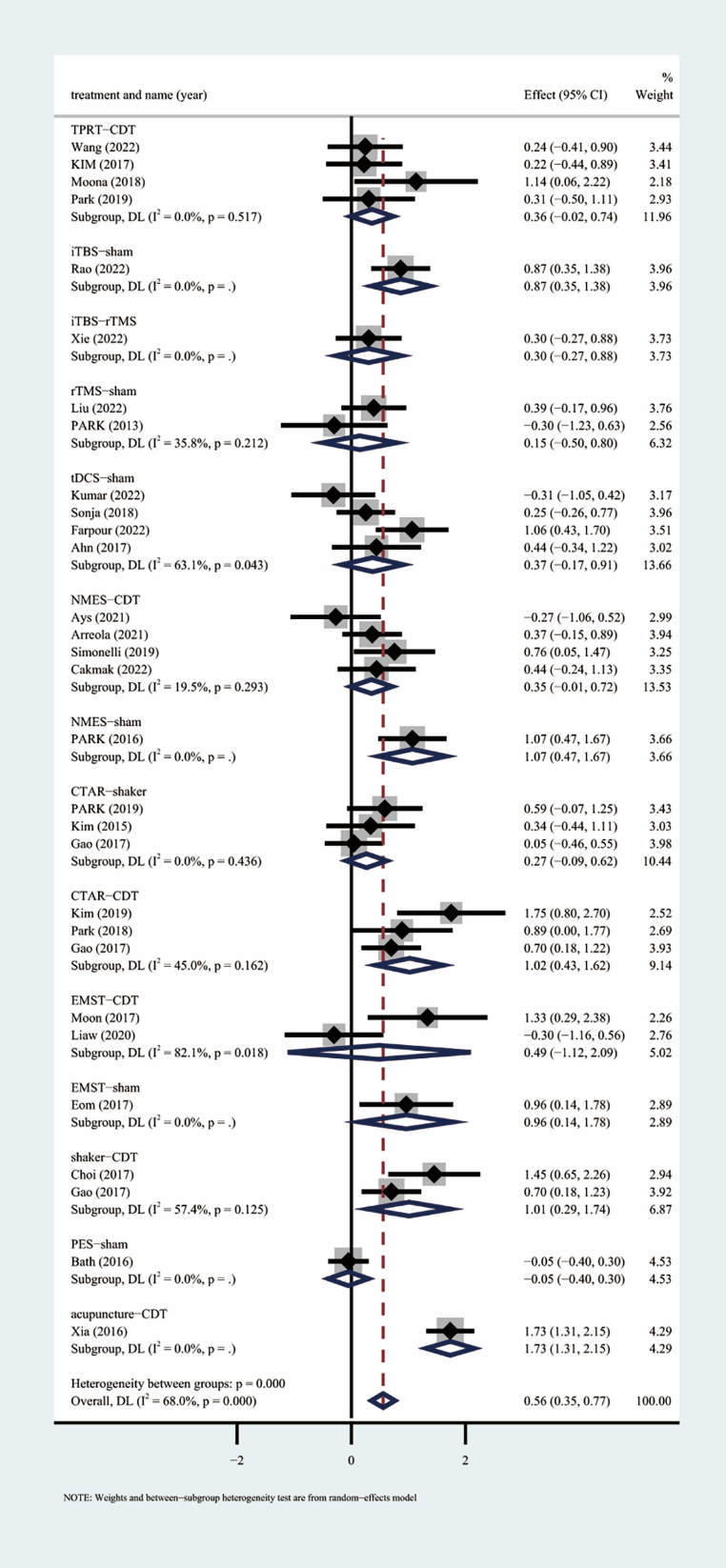


4. Paired meta-analysis forest of feeding and daily function


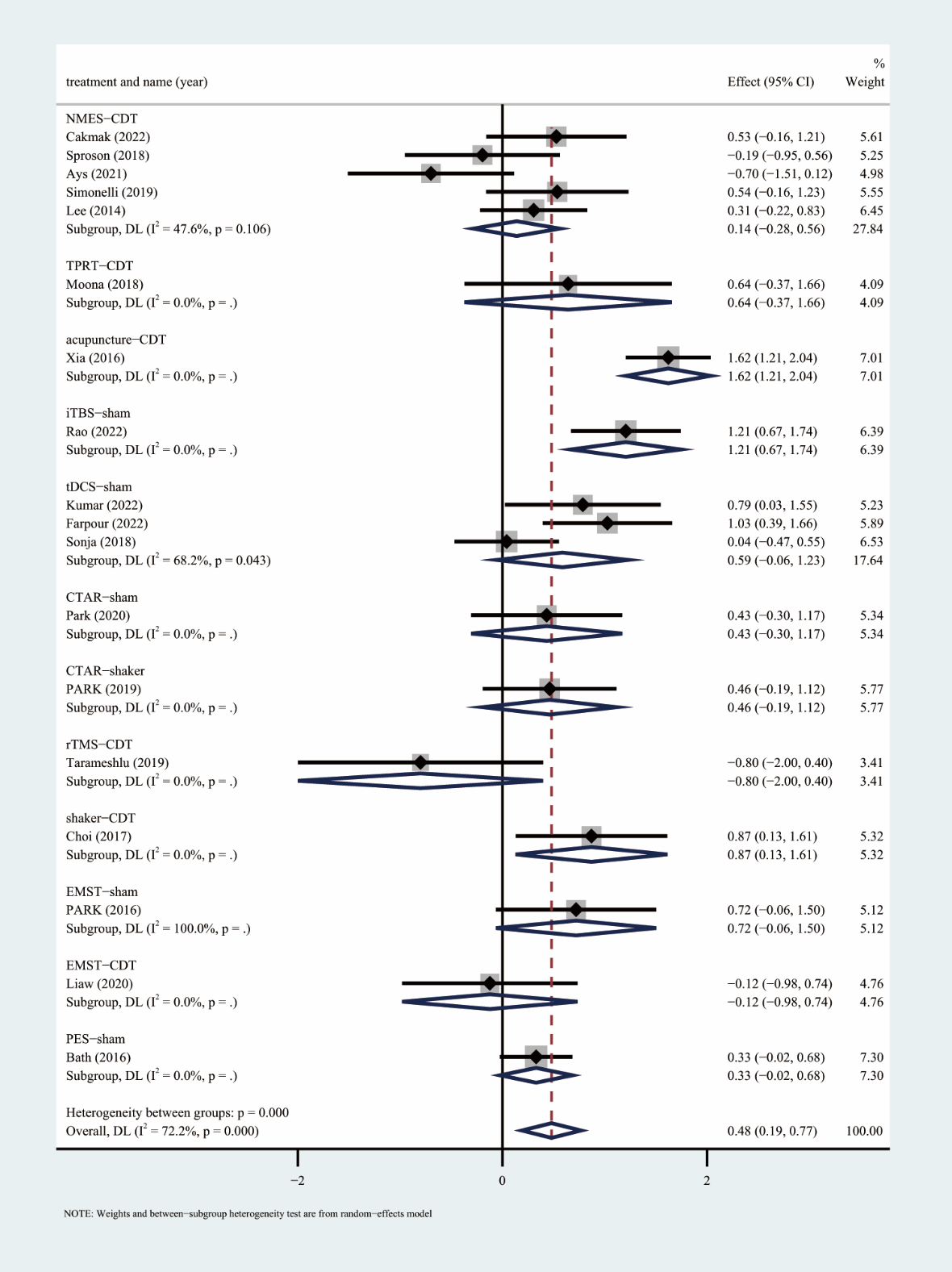


5. Network meta-analysis forest of swallowing function


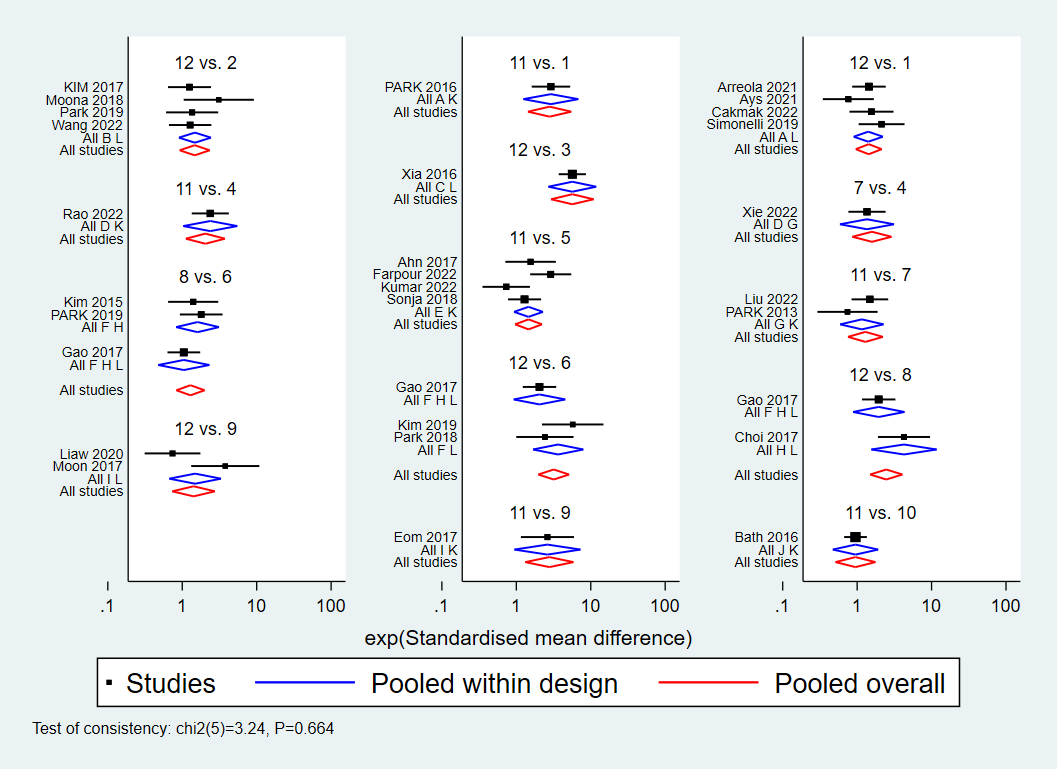


Abbreviations: 1: Neuromuscular electrical stimulation; 2: Tongue-pressure resistance training; 3: acupuncture; 4: Intermittent theta burst stimulation; 5: Transcranial direct current stimulation; 6: Chin Tuck against resistance exercise; 7: Repetitive transcranial magnetic stimulation; 8: shaker; 9: Expiratory muscle strength training; 10: Pharyngeal electric stimulation; 11: sham control; 12: Conventional dysphagia training

6. Network meta-analysis forest of feeding and daily function


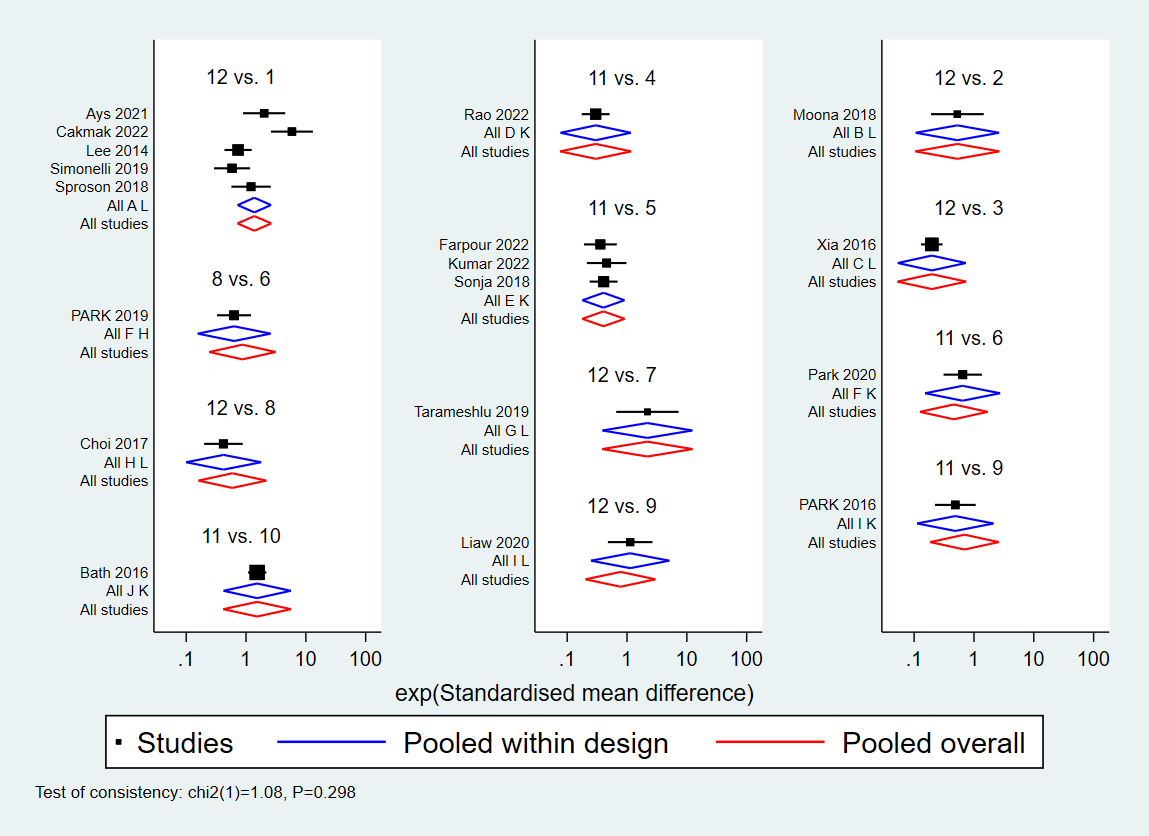


Abbreviations: 1: Neuromuscular electrical stimulation; 2: Tongue-pressure resistance training; 3: acupuncture; 4: Intermittent theta burst stimulation; 5: Transcranial direct current stimulation; 6: Chin Tuck against resistance exercise; 7: Repetitive transcranial magnetic stimulation; 8: shaker; 9: Expiratory muscle strength training; 10: Pharyngeal electric stimulation; 11: sham control; 12: Conventional dysphagia training

7. Network meta-analysis of intervalplot of swallowing function


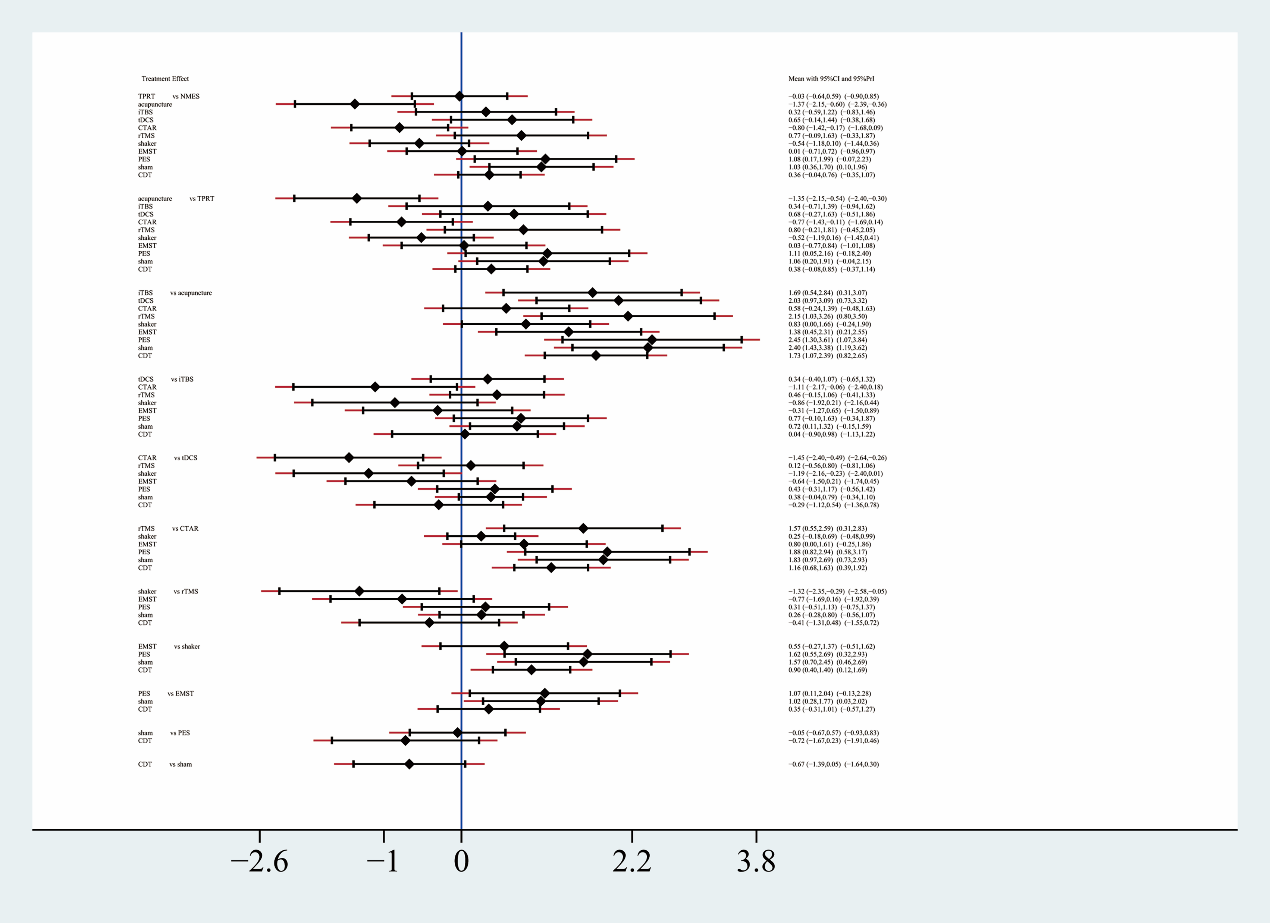


8. Network meta-analysis intervalplot of feeding and daily function


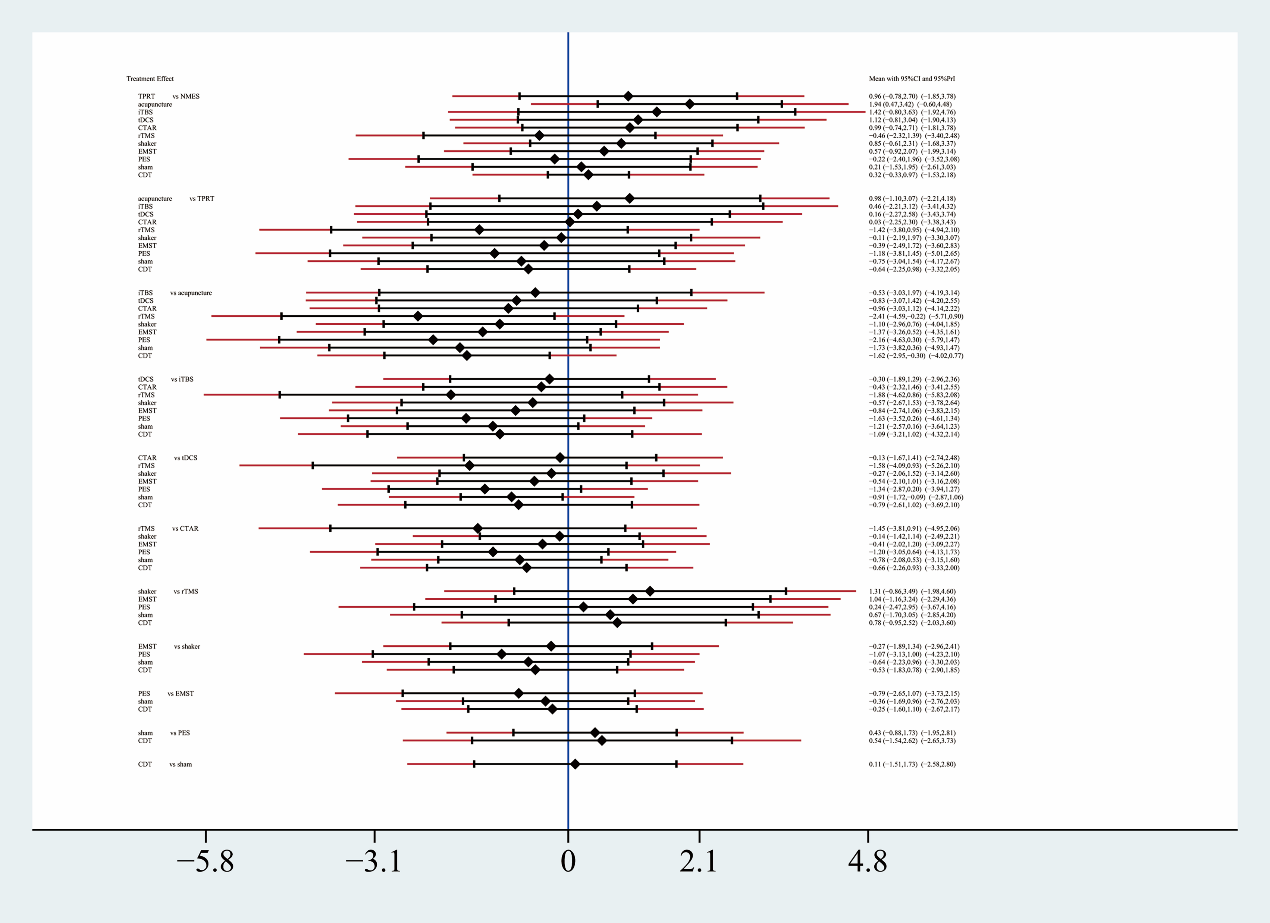


9. Publication bias of swallowing function


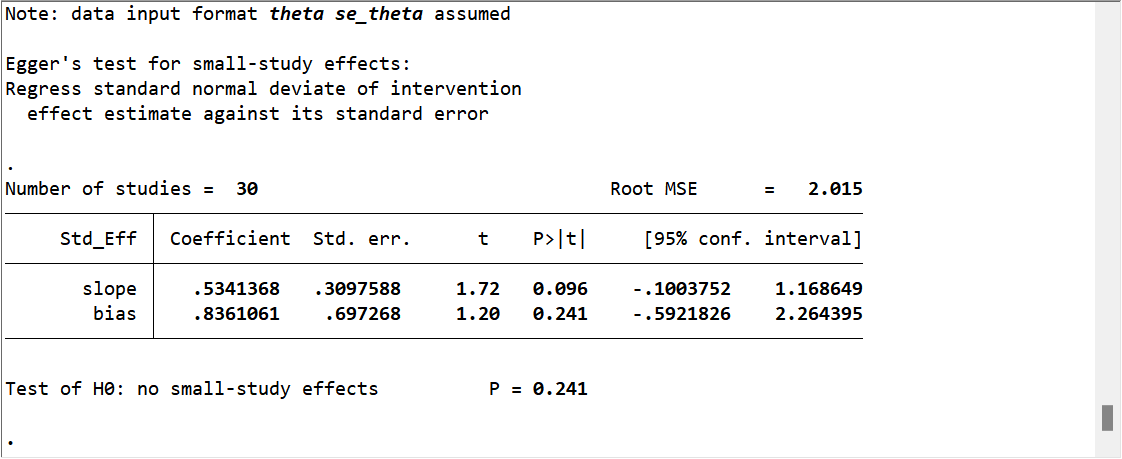


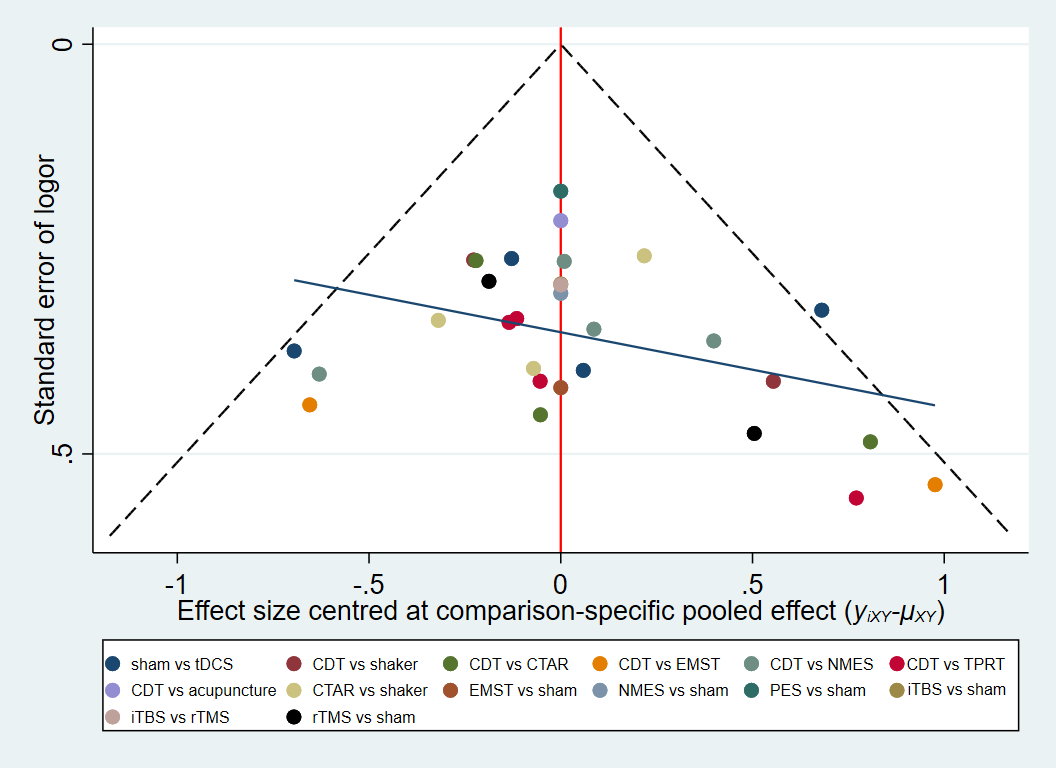


10. Publication bias of feeding and daily function


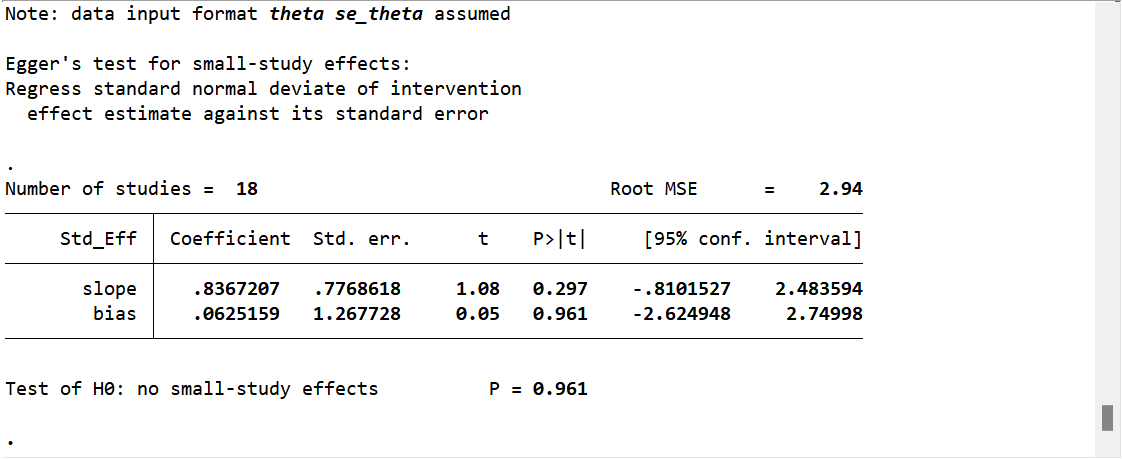


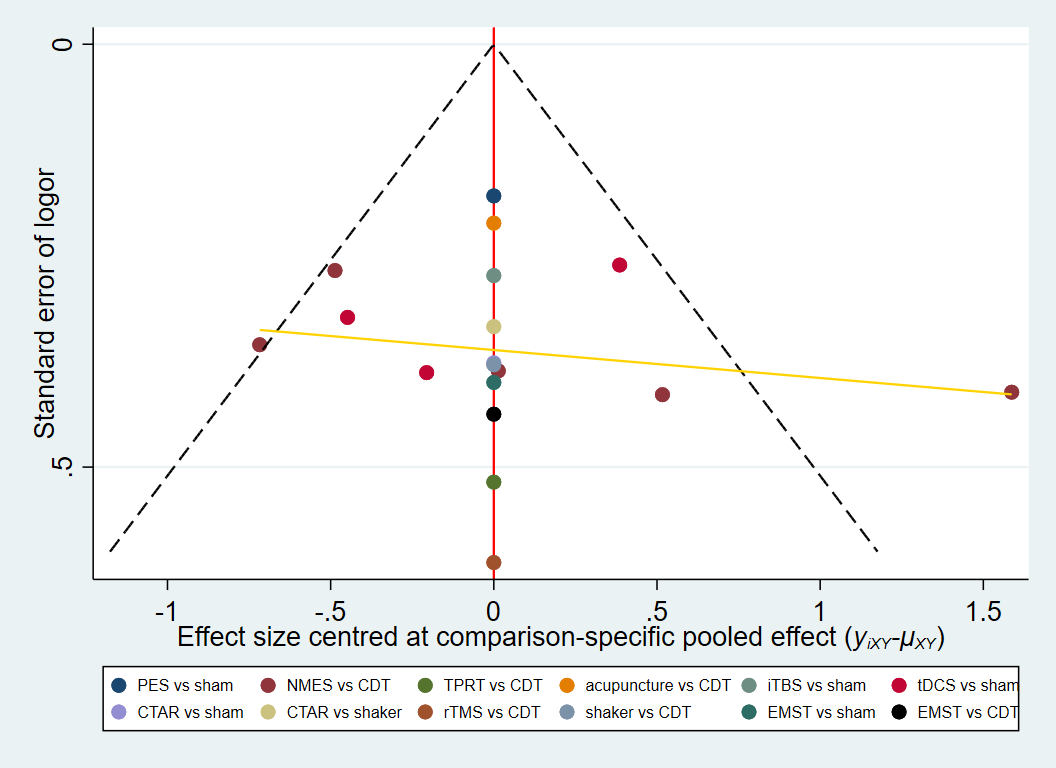


11. Sensitivity analysis of swallowing function


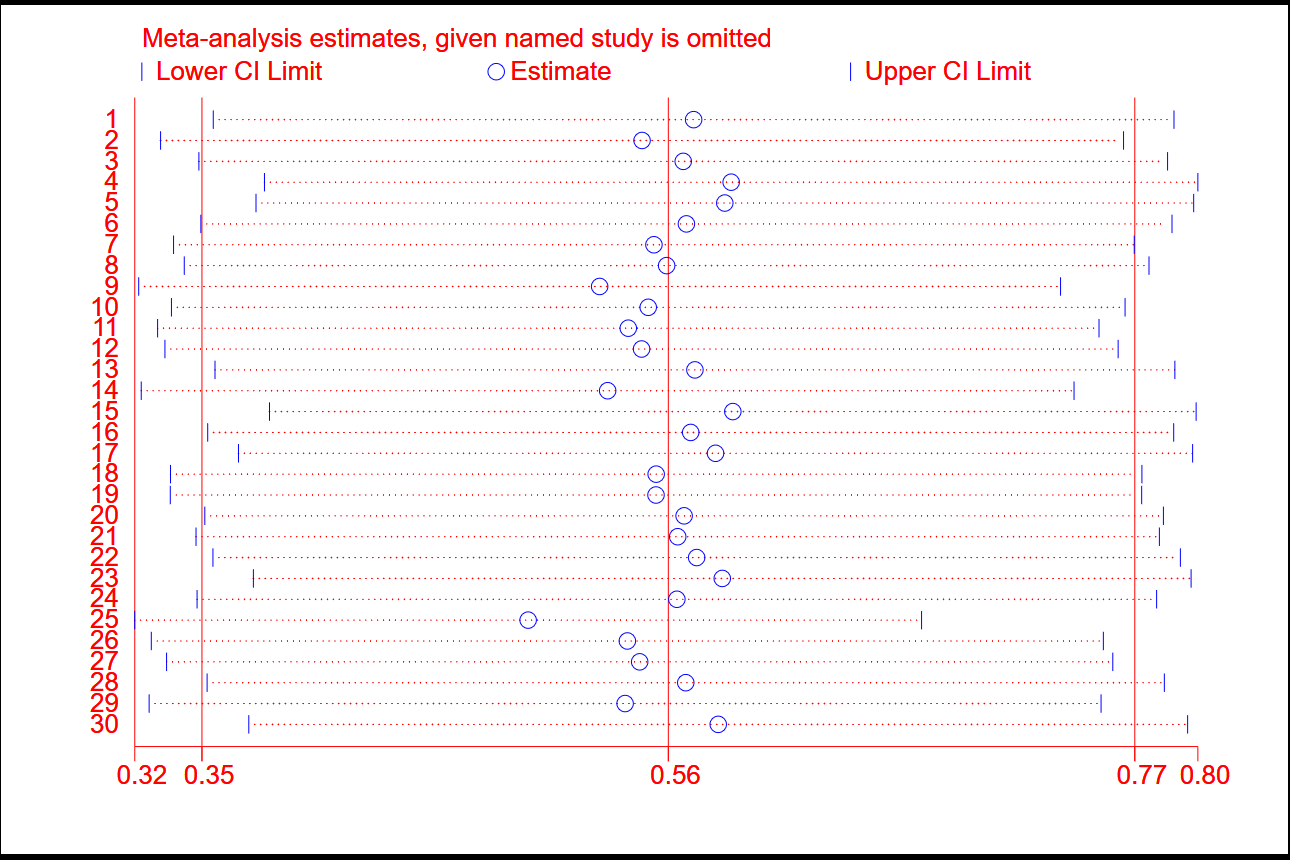


12. Sensitivity analysis of feeding and daily function


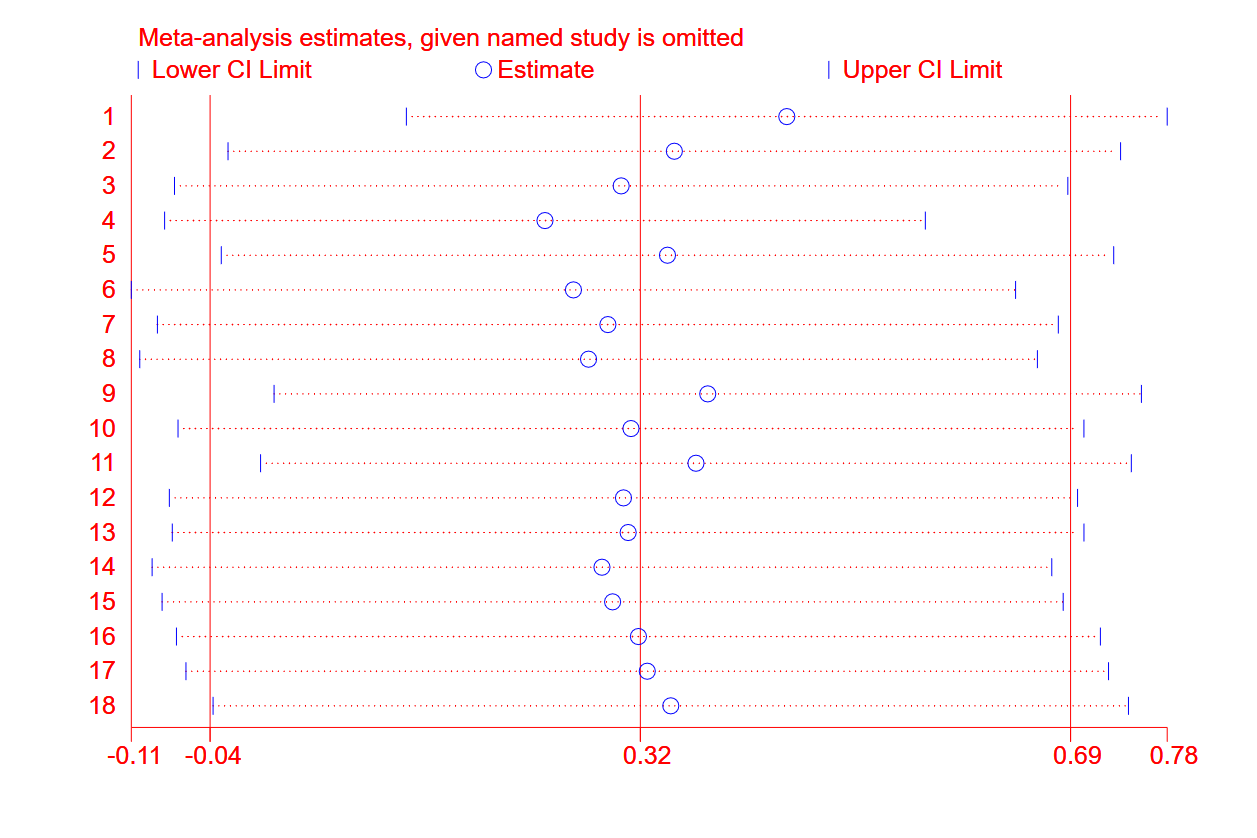

Supplement: Supplementary file 4 — Supporting Information S4. [file JOOR-52-109-s001.docx]
